# Inosine is an alternative carbon source for CD8^+^-T-cell function under glucose restriction

**DOI:** 10.1038/s42255-020-0219-4

**Published:** 2020-06-15

**Authors:** Tingting Wang, J. N. Rashida Gnanaprakasam, Xuyong Chen, Siwen Kang, Xuequn Xu, Hua Sun, Lingling Liu, Hayley Rodgers, Ethan Miller, Teresa A. Cassel, Qiushi Sun, Sara Vicente-Muñoz, Marc O. Warmoes, Penghui Lin, Zayda Lizbeth Piedra-Quintero, Mireia Guerau-de-Arellano, Kevin A. Cassady, Song Guo Zheng, Jun Yang, Andrew N. Lane, Xiaotong Song, Teresa W.-M. Fan, Ruoning Wang

**Affiliations:** 1 Center for Childhood Cancer & Blood Diseases, Hematology/Oncology & BMT, Abigail Wexner Research Institute at Nationwide Children’s Hospital, Ohio State University, Columbus, OH USA; 2 The Center for Cell and Gene Therapy, Baylor College of Medicine, Houston, TX USA; 3 Center for Environmental and Systems Biochemistry, Department of Toxicology and Cancer Biology, Markey Cancer Center, University of Kentucky, Lexington, KY USA; 4 School of Health and Rehabilitation Sciences, Division of Medical Laboratory Science, College of Medicine, Wexner Medical Center, Ohio State University, Columbus, OH USA; 5 Division of Rheumatology and Immunology, Department of Internal Medicine at Ohio State University of Medicine and Wexner Medical Center, Columbus, OH USA; 6 Department of Surgery, St Jude Children’s Research Hospital, Memphis, TN USA; 7 Icell Kealex Therapeutics, Houston, TX USA

**Keywords:** Cytotoxic T cells, Metabolomics, Metabolic pathways, Cancer

## Abstract

T cells undergo metabolic rewiring to meet their bioenergetic, biosynthetic and redox demands following antigen stimulation. To fulfil these needs, effector T cells must adapt to fluctuations in environmental nutrient levels at sites of infection and inflammation. Here, we show that effector T cells can utilize inosine, as an alternative substrate, to support cell growth and function in the absence of glucose in vitro. T cells metabolize inosine into hypoxanthine and phosphorylated ribose by purine nucleoside phosphorylase. We demonstrate that the ribose subunit of inosine can enter into central metabolic pathways to provide ATP and biosynthetic precursors, and that cancer cells display diverse capacities to utilize inosine as a carbon source. Moreover, the supplementation with inosine enhances the anti-tumour efficacy of immune checkpoint blockade and adoptive T-cell transfer in solid tumours that are defective in metabolizing inosine, reflecting the capability of inosine to relieve tumour-imposed metabolic restrictions on T cells.

## Abstract (web summary)

Lymphocytes encounter fluctuations in nutrient availability at sites of infection and inflammation. Wang et al. report that inosine can fulfil the metabolic needs of glucose-restricted anti-tumour effector CD8^+^ T cells.

## Main

A robust adaptive immune response relies on the ability of antigen-specific T cells to rapidly transform from a quiescent (naive) state to a proliferative (active) state, followed by sustained proliferation during the period of antigen presentation. An increasing body of evidence suggests that a coordinated rewiring of cellular metabolism is required to fulfil the bioenergetic, biosynthetic and reduction–oxidation (redox) demands of T cells following activation^1–7^. Naive T cells and memory T cells predominantly rely on fatty acid β-oxidation (FAO) and oxidative phosphorylation for their energy supply in the quiescent state. Following antigen stimulation, effector T (T_eff_) cells rapidly upregulate other pathways, including aerobic glycolysis, the pentose phosphate pathway (PPP) and glutaminolysis, to drive clonal expansion and effector functions^8–11^. Such metabolic reprogramming induced by T-cell activation relies on a hierarchical signalling cascade that links to transcriptional networks^8,10,12^.

It is now commonly recognized that the immune system is intimately involved in tumour initiation, progression and responses to therapy^13–15^. T_eff_ cells are the major agents that elicit anti-tumour activity through directly recognizing and killing antigen-presenting tumour cells, as well as orchestrating a plethora of adaptive and innate immune responses^14,16,17^. However, tumours often co-opt a broad spectrum of mechanisms that foster an immunosuppressive microenvironment, thereby escaping T-cell-mediated anti-tumour immune response. Various intrinsic and extrinsic tumour factors favour the development of immunosuppressive myeloid-derived suppressor cells (MDSCs) and regulatory T cells, increasing the expression of inhibitory checkpoint receptors, such as cytotoxic T-lymphocyte-associated protein 4 (CTLA-4), programmed cell death protein-1 (PD-1) and lymphocyte-activation gene 3 (LAG-3), while reducing the expression and presentation of tumour-specific antigens^18–20^.

Mounting evidence has shown that immunotherapies, through strengthening the amplitude and quality of the T-cell-mediated adaptive response, may mediate durable and even complete tumour regression in some people with cancer. Two of the most promising approaches to enhance therapeutic anti-tumour immunity are immune checkpoint blockade using monoclonal antibodies against PD-1/PDL1 and CTLA4, and adoptive cell transfer of tumour-infiltrating lymphocytes (TILs) or peripheral T cells that are genetically engineered with chimaeric antigen receptors (CARs)^21–23^. Despite the clinical promise of CAR-T-cell therapy in B-cell leukaemia and of checkpoint-blockade therapies in metastatic melanoma, non-small-cell lung cancer (NSCLC), bladder cancer and other types of cancer, more than three-quarters of people with cancer overall remain refractory to the current immunotherapeutic regimen^23,24^.

To enhance immunotherapeutic efficacy, understanding the key immunosuppressive barriers in cancer tissues is critically important. A key but largely overlooked problem is nutrient competition between tumour cells and T cells in the nutrient-poor tumour microenvironment (TME). Metabolic dysregulation is now recognized as one of the hallmarks of human cancer, and of the activation of oncogenic signalling pathways that enable tumour cells to reprogram nutrient-acquisition and nutrient-metabolism pathways to meet the additional bioenergetic, biosynthetic and redox demands of cell transformation and proliferation^25–27^. The TME of solid tumours represents a dramatic example of metabolic stress, wherein the high metabolic demands of cancer cells can restrict the function of T_eff_ cells through competition for nutrients, including glucose, and by producing immunosuppressive metabolites^28–32^. As such, a better understanding of the metabolic modulation of T_eff_ cells that relieves the immunosuppressive barriers in the TME will enable us to devise rational and effective approaches to enhance cancer immunotherapy via improvement of the metabolic fitness of T cells.

Here, we report that T cells can utilize few substrates, and that inosine is an alternative metabolic substrate that can support cell growth and crucial T-cell functions in the absence of glucose in vitro. The catabolism of the ribose subunit of inosine provides both metabolic energy in the form of ATP and biosynthetic precursors from glycolysis and the PPP. Inosine supplementation promotes T-cell-mediated tumour-killing activity in vitro and enhances the anti-tumour efficacy of checkpoint-blockade therapy or adoptive T-cell therapy in certain mouse models, reflecting the capability of inosine in relieving tumour-imposed metabolic restrictions on T cells.

## Results

### A metabolic screen identifies inosine as an alternative fuel for T cells in vitro

**Fig. 1:**
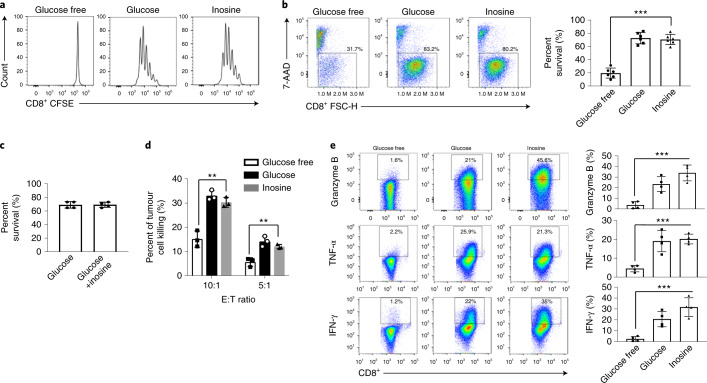
Inosine can support proliferation and function of mouse T_eff_ cells in the absence of glucose. Naive CD8^+^ T cells from C57BL/6 mice were activated by plate-bound anti-CD3 and anti-CD28 antibodies in complete medium for 24 h, and then the cells were switched to the indicated conditioned media (either glucose free, containing glucose or containing inosine) and were cultured for 48 h. **a**,**b**, Cell proliferation (**a**) and cell death (**b**) were determined by carboxyfluorescein succinimidyl ester (CFSE) dilution and 7-aminoactinomycin D (7-AAD) uptake, respectively. Data are presented as mean ± s.d. (*n* = 6). ***P = 4 × 10^–7^ for glucose free versus inosine. Data are representative of three independent experiments. M denotes million. **c**, Mouse T_eff_ cells were cultured in glucose-containing medium in the presence or absence of inosine for 72 h, and cell survival was determined by 7-AAD uptake. Data are presented as mean ± s.d. (*n* = 4). Data are representative of three independent experiments. **d**, Splenocytes from Pmel transgenic mice were isolated and cultured with 1 μM human gp100 and 30 U ml^–1^ recombinant human IL-2 in complete medium for 4 d, and were switched to the indicated conditioned media for 72 h. B16 melanoma cells were co-cultured with activated Pmel T cells, and the percentage of tumour-cell lysis was determined by calcein release with the Spectramax M2 microplate reader. Data are presented as mean ± s.d. (*n* = 3). **P = 0.0025 and 0.0053 for effector to target (E:T) ratio 10:1 and 5:1 for glucose free versus inosine, respectively. **e**, Naive CD8^+^ T cells from C57BL/6 mice were activated by plate-bound anti-CD3 and anti-CD28 antibodies and were differentiated in the indicated conditioned media for 4 d. The indicated proteins were quantified by intracellular staining following phorbol 12-myristate 13-acetate (PMA) and ionomycin stimulation. Data are presented as mean ± s.d. (*n* = 4). ***P = 0.0003, 0.00005, 0.0006 for granzyme B, TNF-α and IFN-γ for glucose free versus inosine, respectively. Data were analysed by unpaired two-sided *t*-test (**b**,**d**,**e**). Sample size (*n*) represents biologically independent samples (**b–e**). Source data

**Extended Data Fig. 1:**
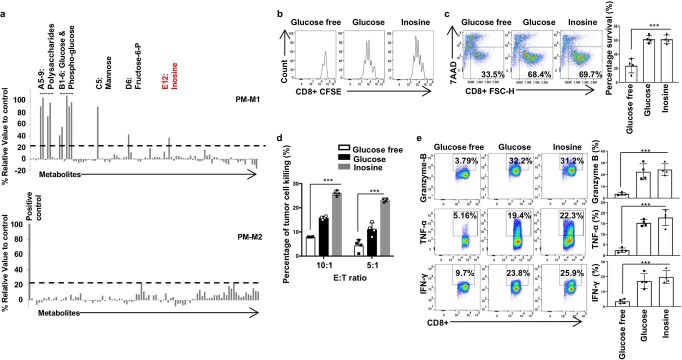
Inosine can support human T effector cell proliferation and function in the absence of glucose. **a**, Metabolite array from the PM-M1 and PM-M2 Microplates (BioLog Inc). PBMC cells were stimulated with plate-bound anti-CD3 antibody for at least three days to generate active human T cells. Cells were then collected and incubated with various energy sources in a PM-M1 or PM-M2 microplate for 24 h, followed by Biolog redox dye mix MB incubation and measured spectrophotometrically at 590 nm. **b**, **c**, Naive CD8+ T cells from human PBMC were activated by plate-bound anti-CD3 and anti-CD28 antibodies in the indicated conditional media for 5 days. Cell proliferation and cell death of active CD8+ T cells were determined by CFSE dilution and 7AAD uptake, respectively. Data are presented as mean ± SD (n=4). ***, *P*=0.0007 for Glucose free versus Inosine. **d**, GD2-CAR T cells are generated using complete media, then switched to indicated conditional media and cultured for 48-72 h. LAN-1 cells were co-cultured with GD2-CAR T cells in the indicated conditional media, and cell death of LAN-1 was determined by calcein release with the Spectramax M2 microplate reader. Data are presented as mean ± SD (n=4). ***, *P*=5E-8 and 0.000006 for E:T ratio 10:1 and 5:1 for Glucose free versus Inosine respectively. **e**, The indicated proteins in GD2-CAR T cells were quantified by intracellular staining following PMA and ionomycin stimulation. Data are presented as mean ± SD (n=4). ***, *P*=0.0002, 0.0002, 0.0004 for Granzyme B, TNF-α, and IFN-γ for Glucose free versus Inosine, respectively. Data were analyzed by unpaired two-sided t-test (**c**–**e**). Sample size (n) represents biologically independent samples (**c**–**e**). Source data

Solid tumours typically develop a hostile microenvironment that is characterized by an irregular vascular network and a correspondingly poor nutrient supply. Highly glycolytic cancer cells may deplete nutrients, particularly glucose, thereby restricting glucose availability to T cells^28–30^. Previous studies have shown that murine T cells substantially enhanced glucose catabolism following activation, and that glucose starvation compromises the viability and proliferation of T_eff_ cells^8,10,33^. We asked whether T_eff_ cells have the capability to take up alternative carbon and energy sources to support their survival and proliferation in the absence of glucose. To this end, we generated human T_eff_ cells by stimulating human peripheral blood mononuclear cells (PBMCs) with plate-bound anti-CD3 antibody in the presence of interleukin 2 (IL-2). After at least 3 d of activation and expansion of human T_eff_ cells, we plated the cells in glucose-free medium into 96-well plates (Biolog’s Phenotype MicroArray Mammalian plates PM-M1 and PM-M2) that were pre-loaded with an array of compounds that can serve as carbon and/or nitrogen sources for mammalian cells. Blank wells and wells pre-loaded with glucose were included as negative and positive controls, respectively. We then determined the substrate utilization of T_eff_ cells in each well through a colorimetric assay in which the formation of the reduced dye represents the cells’ ability to catabolize the extracellular substrate and generate NADH. The results were normalized to positive controls (wells pre-loaded with glucose) and are summarized in Extended Data Fig. 1a. We found that polysaccharides and six-carbon sugars and their derivatives, including dextrin, glycogen, maltotriose, maltose, mannose and fructose 6-phosphate, were utilized by T_eff_ cells in the absence of glucose (Extended Data Fig. 1a). Remarkably, inosine, a nucleoside, also supported T_eff_ cells’ bioenergetic activity in the absence of glucose (Extended Data Fig. 1a). To eliminate the possibility that inosine was contaminated with glucose or glucose analogues, we obtained inosine from a different source and examined whether inosine could support proliferation and viability of mouse and human T_eff_ cells in the absence of glucose. Glucose starvation significantly reduced cell proliferation and increased cell death (Fig. 1a, b and Extended Data Fig. 1b,c). When supplemented at an equimolar amount of glucose, inosine markedly reduced cell death and restored cell proliferation in mouse and human T_eff_ cells following glucose starvation (Fig. 1a,b and Extended Data Fig. 1b,c). However, inosine supplementation did not further enhance T-cell viability in the glucose-containing medium (Fig. 1c). Together, our results show that inosine may offer bioenergetic support as glucose does to support proliferation of T_eff_ cells in vitro.

### Inosine supports the function of T_eff_ cells in the absence of glucose in vitro

We next asked whether inosine could restore the cytotoxic activity and other effector functions of T_eff_ cells in the absence of glucose. We generated antigen-specific mouse T_eff_ cells by stimulating major histocompatibility complex (MHC)-class-I-restricted T cells, isolated from premelanosome protein-1 (Pmel-1) T-cell-receptor transgenic mice, with peptide antigen and IL-2. We also generated human GD2-specific CAR (GD2-CAR) T cells using PBMCs by retroviral transduction with GD2-CAR. We found that Pmel^+^ cells recognized Pmel-17 (mouse homologue of human PMEL (also known as gp100)) in mouse melanoma, and GD2-CAR T cells recognized GD2, a disialoganglioside expressed in tumours of neuroectodermal origin. Mouse Pmel^+^ T_eff_ cells or human anti-GD2-CAR T cells were cultured in glucose-free, glucose-containing or inosine-containing medium for 48–72 h. We then co-cultured Pmel^+^ T_eff_ cells with gp100-expressing B16 mouse melanoma cells (B16-gp100; Fig. 1d), and we co-cultured GD2-CAR T cells with LAN-1 human neuroblastoma cells (Extended Data Fig. 1d), in conditioned medium for 4 h to minimize the impact of the conditioned medium on tumour-cell viability and proliferation, and the killing of tumour cells was assessed^34^. Inosine supplementation restored tumour killing activities in mouse and human T_eff_ cells following glucose starvation (Fig. 1d and Extended Data Fig. 1d). We also cultured mouse (Fig. 1e) and human (Extended Data Fig. 1e) T_eff_ cells in conditioned medium and assessed the expression of effector molecules, including granzyme B, tumour necrosis factor alpha (TNF-α) and interferon gamma (IFN-γ), all of which are important in mediating the tumour-cell-killing activity of T_eff_ cells. Although glucose starvation significantly reduced the tumour-killing capacity and the expression of granzyme B, TNF-α and IFN-γ of T_eff_ cells, both glucose and inosine supplementation restored these properties in mouse and human T_eff_ cells (Fig. 1d,e and Extended Data Fig. 1d,e). Taken together, our results indicate that inosine has the capacity to replace glucose in supporting the effector function of T_eff_ cells in vitro.

### Adenosine cannot support the function of T_eff_ cells in the absence of glucose

**Extended Data Fig. 2:**
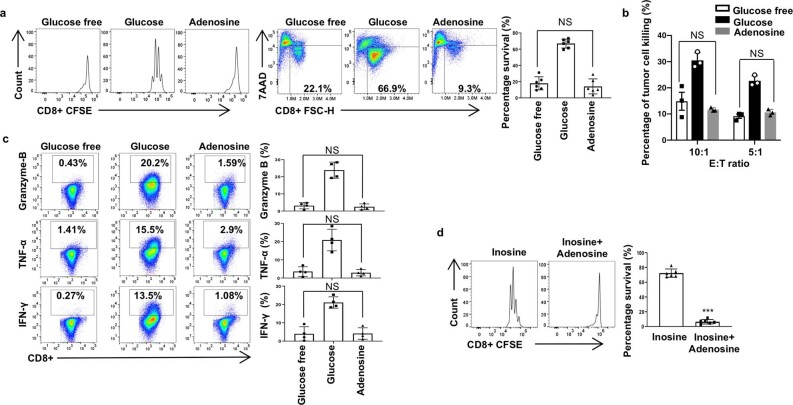
Adenosine cannot support mouse T effector cell proliferation and function in the absence of glucose. **a**, Naive CD8+ T cells from C57BL/6 mice were activated by plate-bound anti-CD3 and anti-CD28 antibodies in the indicated conditional media for 3 days. Cell proliferation and cell death of active CD8+ T cells were determined by CFSE dilution and 7AAD uptake, respectively. Data are presented as mean ± SD (n=6). NS, *P*=0.4668 for Glucose free versus Adenosine. **b**, B16 melanoma cells were co-cultured with activated Pmel T cell that are generated in complete media followed by 72 h incubation in the indicated conditional media and the percentage of tumor cell lysis was determined by calcein release with the Spectramax M2 microplate reader. Data are presented as mean ± SD (n=4). NS, *P*=0.4107 and 0.2385 for E:T ratio 10:1 and 5:1 for Glucose free versus Adenosine, respectively. **c**, Naive CD8+ T cells from C57BL/6 mice were activated by plate bound anti-CD3 and anti-CD28 antibodies and differentiated in the indicated conditional media for 4 days. The indicated proteins were quantified by intracellular staining following PMA and ionomycin stimulation. Data are presented as mean ± SD (n=4). NS, *P*=0.6305, 0.6906, 0.9495 for Granzyme B, TNF-α, and IFN-γ for Glucose free versus Adenosine, respectively. **d**, Naive CD8+ T cells from C57BL/6 mice were activated in the indicated conditional media for 3 days. Cell proliferation and cell death of active CD8+ T cells were determined by CFSE dilution and 7AAD uptake, respectively. Data are presented as mean ± SD (n=6). ***, *P*=2E-10 for Inosine versus Inosine+Adenosine. Data were analyzed by unpaired two-sided t-test (**a**–**d**). Sample size (n) represents biologically independent samples (**a**-**d**). Source data

**Extended Data Fig. 3:**
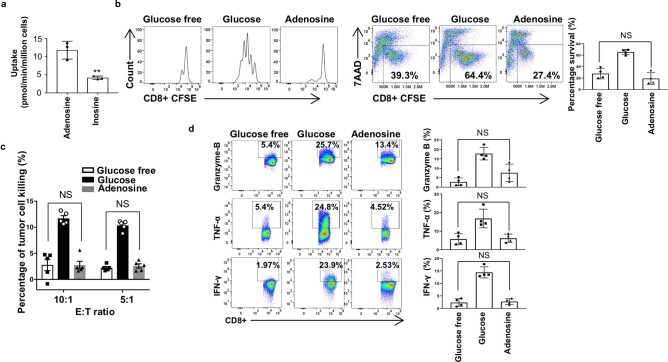
Adenosine cannot support human T effector cell proliferation and function in the absence of glucose. **a**, Human PBMCs were stimulated with plate-bound anti-CD3 and anti-CD28 antibodies for at least three days to generate active human T cells. Uptake of adenosine or inosine in active human T cells was determined by the cellular retention of corresponding radioactive tracers. Data are presented as mean ± SD (n=3). Data are representative of two independent experiments. **, *P*=0.0063 for Adenosine versus Inosine. **b**, Naive CD8+ T cells from PBMCs were activated by plate-bound anti-CD3 and anti-CD28 antibodies in the indicated conditional media for 5 days. Cell proliferation and cell death of active CD8+ T cells were determined by CFSE dilution and 7AAD uptake, respectively. Data are presented as mean ± SD (n=4). NS, *P*=0.2585 for Glucose free versus Adenosine by unpaired two-sided t-test. **c**, GD2-CAR T cells are generated using complete media, then switched to indicated conditional media and cultured for 72 h. LAN-1 cells were co-cultured with GD2-CAR T cells in the indicated conditional media, and cell death of LAN-1 was determined by calcein release. Data are presented as mean ± SD (n=4). NS, *P*=0.9764 and 0.4716 for E:T ratio 10:1 and 5:1 for Glucose free versus Adenosine, respectively. **d**, The indicated proteins in GD2-CAR T cells were quantified by intracellular staining following PMA and ionomycin stimulation. Data are presented as mean ± SD (n=4). NS, *P*=0.1058, 0.8182, 0.7081 for Granzyme B, TNF-α, and IFN-γ for Glucose free versus Adenosine, respectively. Data were analyzed by unpaired two-sided t-test (**a**–**d**). Sample size (n) represents biologically independent samples (**a**–**d**). Source data

Adenosine, the metabolic precursor of inosine, has been reported to be an immune-suppressive metabolite, and it promotes the resolution of inflammation by targeting a range of immune cells, including T cells^35,36^. Adenosine and inosine have identical chemical properties, and active human T cells are able to take up adenosine at a higher rate than they can take up inosine (Extended Data Fig. 3a). Thus, we asked whether adenosine can support the proliferation of T_eff_ cells in the absence of glucose. Adenosine, supplemented at an equimolar amount of glucose, failed to reduce cell death or restore the proliferation of mouse and human T_eff_ cells following glucose starvation (Extended Data Figs. 2a and 3b). Next, we asked whether adenosine can support the effector functions of T_eff_ cells in the absence of glucose. We found that adenosine failed to restore the tumour-killing activity and the expression of effector molecules in mouse (Extended Data Fig. 2b,c) and in human (Extended Data Fig. 3c,d) T_eff_ cells in the absence of glucose. Furthermore, adenosine supplementation could suppress inosine-dependent T_eff_-cell proliferation and survival (Extended Data Fig. 2d). Collectively, our results indicate that inosine, but not its metabolic precursor adenosine, has the capacity for replacing glucose to support the cytotoxic function of T_eff_ cells in vitro.

**Extended Data Fig. 4:**
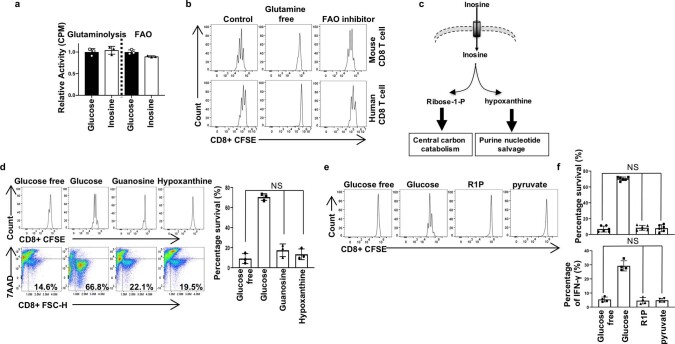
Inosine does not support T effector cell proliferation by enhancing glutamine and fatty acids catabolism or through providing purine catabolites. **a**, Naive CD8+ T cells from C57BL/6 mice were activated by plate-bound anti-CD3 and anti-CD28 antibodies in complete media for 24 h, then the cells were switched to indicated conditional media and were cultured for 24 h. The rates of glutaminolysis and fatty acid β-oxidation (FAO) were measured by the generation of ^14^CO_2_ from [U-^14^C]-glutamine and ^3^H_2_O [9, 10-^3^H]-palmitic acid, respectively. Error bars represent standard deviation from the mean of triplicate samples (n=3). Data are representative of two independent experiments. **b**, Naive CD8+ T cells from C57BL/6 mice (upper) and human PBMCs (lower) were activated by plate-bound anti-CD3 and anti-CD28 antibodies in complete media for 24 h, then cells were switched to indicated culture condition for 3-5 days. Cell proliferation of active CD8+ T cells was determined by CFSE dilution. Data are representative of two independent experiments. **c**, Diagram of inosine uptake and breakdown into ribose 1-phosphate (Ribose-1-P) and hypoxanthine, entering central carbon catabolism and the purine nucleotide salvage pathway, respectively. (**d**–**f**) Naive CD8+ T cells from C57BL/6 mice were cultured in the indicated conditional media for 72 h. Cell proliferation and cell death were determined by CFSE dilution and 7AAD uptake, respectively. The indicated protein in mouse CD8+ T cells were quantified by intracellular staining following PMA and ionomycin stimulation. Data are presented as mean ± SD (n=3, 6, 4 for **e**, **f** (upper), **f** (lower)). **d**, NS, *P*=0.1505 and 0.3749 for Glucose free versus Guanosine and Hypoxanthine, respectively. **f**, NS, *P*=0.4329, 0.6512, 0.5853, 0.6022 for Glucose free versus R1P or pyruvate for survival and IFN-γ, respectively. Data are representative of four independent experiments. Data were analyzed by unpaired two-sided t-test (**d**, **f**). Sample size (n) represents biologically independent samples (**a**–**f**). Source data

Glutamine is a key carbon and nitrogen donor for T_eff_ cells, and fatty acids are also known carbon donors for naive T, regulatory T and memory T cells^3,5,37^. We next asked whether inosine supports T_eff_-cell proliferation by enhancing glutaminolysis and FAO. Mouse T_eff_ cells cultured in conditioned medium containing either glucose or inosine displayed comparable catabolic activities via glutaminolysis, as indicated by ^14^CO_2_ release from [^14^C_5_]glutamine, and via FAO, as indicated by [^3^H]water release from [9,10-^3^H]palmitic acid (Extended Data Fig. 4a). Moreover, an inhibitor of FAO (etomoxir) failed to block T_eff_-cell proliferation in inosine-containing conditioned medium (Extended Data Fig. 4b). However, glutamine starvation blocks inosine-dependent T_eff_-cell proliferation (Extended Data Fig. 4b), supporting the indispensable role of glutamine as a key nitrogen donor of T_eff_ cells^5^. Collectively, our results show that inosine does not enhance glutamine and fatty acid catabolism in T_eff_ cells. Inosine is a nucleoside that can be broken down into ribose-1-phosphate (R1P) and hypoxanthine, the latter of which can funnel into the salvage pathway for purine nucleotides (Extended Data Fig. 4c)^38^. We next asked whether hypoxanthine or the purine nucleoside guanosine can support the proliferation of T_eff_ cells in the absence of glucose. Neither hypoxanthine nor guanosine, supplemented at an equimolar amount of glucose, reduced cell death or restored the proliferation of mouse T_eff_ cells following glucose starvation (Extended Data Fig. 4d). In addition, we assessed whether R1P or pyruvate supplementation had any impact on T cells in the absence of glucose. Neither R1P nor pyruvate could restore T_eff_-cell proliferation, T_eff_-cell viability or the secretion of IFN-γ (Extended Data Fig. 4e,f). Uric acid is a metabolic product of hypoxanthine and has been shown to enhance T-cell effector functions^39,40^. Next, we assessed the effects of supplementation of uric acid on mouse T_eff_-cell viability and proliferation, and on the expression of effector molecules. We found that, although uric acid failed to restore cell viability, proliferation and the expression of effector molecules in the absence of glucose, it moderately increased the percentage of T_eff_ cells expressing IFN-γ and TNF-α in the presence of glucose (Supplementary Fig. 1a–c). Collectively, our results indicate that inosine does not support the proliferation of T_eff_ cells by enhancing glutamine and fatty acid catabolism or through providing hypoxanthine or uric acid.

### Inosine-derived ribose fuels key metabolic pathways in T_eff_ cells in vitro

**Fig. 2:**
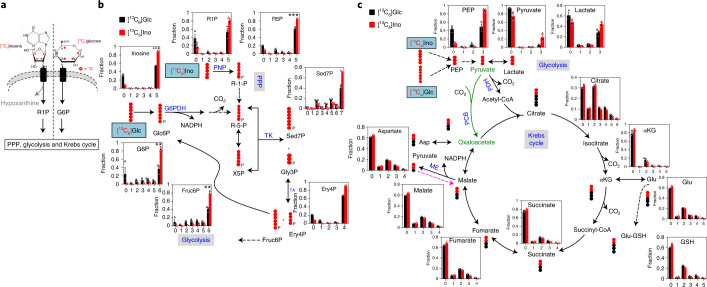
The ribose subunit of inosine can replace glucose and feed into the central carbon metabolism in T_eff_ cells. **a**, Diagram of [^13^C_5_]inosine and [^13^C_6_]glucose uptake and conversion to R1P or G6P, respectively, which subsequently enters downstream metabolic pathways: the PPP, glycolysis and the Krebs cycle. The red diamond denotes uniformly ^13^C-labelled positions of the ribose subunit of inosine and all carbons of glucose. **b**,**c**, Active human T cells were incubated in [^13^C_6_]glucose (Glc) or [^13^C_5_]ribose-inosine (Ino) medium for 24 h, were extracted as described in the Methods and were analysed for PPP metabolites (**b**) and glycolytic/Krebs cycle metabolites (**c**) by IC–UHR-FTMS. Data are presented as mean ± s.e.m. (*n* = 3). **P = 0.0028, 0.0021; ***P = 6 × 10^–7^, 0.0003, for [^13^C_6_]G6P, [^13^C_6_]Fruc6P, [^13^C_5_]Inosine and [^13^C_5_]R5P for [^13^C_6_]glucose versus [^13^C_5_]inosine, respectively, by unpaired two-sided *t*-test. X5P, xylulose-5-phosphate; Gly3P, glyceraldehyde-3-phosphate; G6PDH, glucose-6-phosphate dehydrogenase; Glu-GSH, glutamyl unit of glutathione; PCB, pyruvate carboxylase; ME, malic enzyme; TK, transketolase; TA, transaldolase; black dot, ^12^C; red dot, ^13^C derived either from the PDH- or PCB-initiated Krebs cycle reactions, or from the reverse ME reaction. Solid and dashed arrows represent single- and multi-step reactions, and single and double-headed arrows refer to irreversible and reversible reactions, respectively. Numbers on the *x* axes represent the numbers of ^13^C atoms in the given metabolites. Sample size (*n*) represents biologically independent samples (**b**,**c**). Green text and arrows represent metabolite names and routes in a branched pathway; purple arrows represent a reverse reaction; black text represents metabolite names; blue text represents the names of metabolic enzymes and metabolic pathways. Source data

Next, we asked whether inosine-derived ribose (Extended Data Fig. 4c) could be a fuel for key metabolic pathways involved in cell proliferation and survival. To this end, we applied a stable-isotope-based metabolomics approach to compare metabolic routes of glucose and the ribose subunit in inosine. Specifically, we supplied [^13^C_6_]glucose or [1′,2′,3′,4′,5′-^13^C_5_]inosine, in which only the ribose contains carbon-13, as the metabolic tracer in human T_eff_ cells cultured in glucose-free conditioned medium (Fig. 2a). Then, we followed ^13^C incorporation into intermediate metabolites in the central carbon metabolic pathways, including the PPP, glycolysis and the Krebs cycle.

**Extended Data Fig. 5:**
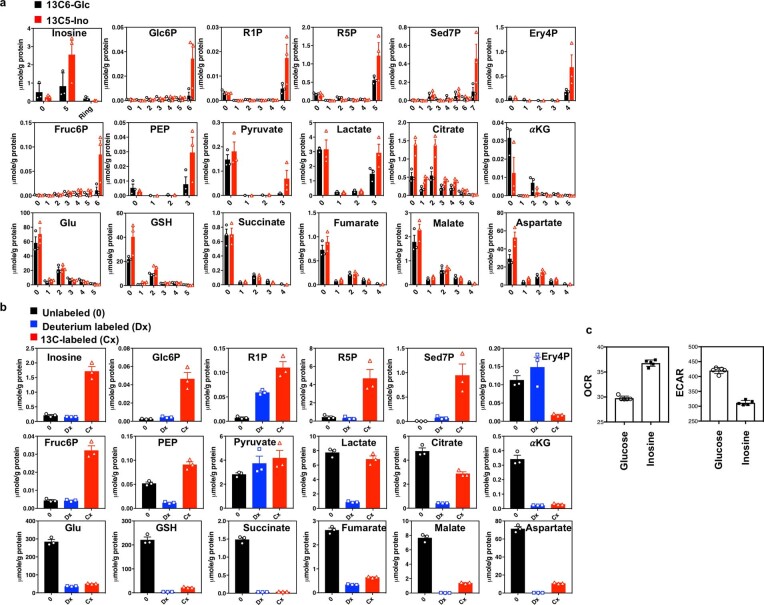
The ribose subunit of inosine can replace glucose and feed into the central carbon metabolism in T effector cells. **a**, The same experiment as in Fig. 2 was performed for active human T cells and the quantification of indicated metabolites were analyzed by IC-UHR-FTMS. Data are presented as mean ± SEM (n=3). Numbers in the X-axis represent those of ^13^C atoms in given metabolites. **b**, Quantification of the metabolites in the dual-tracer experiment with [^13^C_5_]-Inosine and [6,6-D_2_]-Glucose. All symbols and abbreviations are as described in Fig. 2. The X-axis represents unlabeled (0), ^13^C-labeled carbon (Cx), and Deuterium (Dx) in given metabolites. Values represent mean ± SEM (n=3). **c**, OCR/ECAR of active human T cells cultured in glucose versus inosine conditional media. Values represent mean ± SD (n=5). Data are representative of two independent experiments. Sample size (n) represents biologically independent samples (**a**–**c**). Source data

We first compared the metabolism of [^13^C_6_]glucose versus that of [^13^C_5_]inosine via the PPP (Fig. 2b). As expected, the ^13^C_5_ fractional enrichment of the parent tracer inosine was higher in the inosine-tracer-treated T_eff_ cells than in the glucose-tracer-treated ones. Moreover, both tracers were extensively metabolized via the PPP to produce ^13^C-labelled intermediates, including R1P, R5P, sedoheptulose-7-phosphate (Sed7P), erythrose-4-phosphate (Ery4P), fructose-6-phosphate (Fruc6P) and glucose-6-phosphate (G6P) (Fig. 2b and Extended Data Fig. 5a). The fraction of ^13^C enrichment in the dominant fully ^13^C-labelled isotopologues of R5P, Fruc6P and G6P was higher following treatment with the inosine tracer than it was following glucose-tracer treatment. This is consistent with the direct and rapid metabolism of [^13^C_5_]inosine via the non-oxidative branch of the PPP and the equilibration of Fruc6P with G6P via the action of phosphoglucose isomerase (PGI). We also observed the presence of scrambled ^13^C-labelled products of Sed7P ([^13^C_2_]Sed7P and [^13^C_5_]Sed7P, in particular), Fruc6P and G6P. These scrambled products were presumably derived from the reversible transketolase and transaldolase activity. The dominant presence of [^13^C_2_]isotopologues and [^13^C_5_]isotopologues among the scrambled ^13^C-labelled products of Sed7P is again consistent with flux through both branches of PPP for both inosine- and glucose-tracer treatments.

The [^13^C]Fruc6P and [^13^C]G6P products of [^13^C_5_]inosine from PPP can be further metabolized via glycolysis and the Krebs cycle, as in the case of [^13^C_6_]glucose. We thus tracked the fate of [^13^C_5_]inosine and [^13^C_6_]glucose in human T_eff_ cells through these two key bioenergetic pathways via ion chromatography–ultra-high-resolution Fourier transform mass spectrometry (IC–UHR-FTMS) analysis (Fig. 2c and Extended Data Fig. 5a). [^13^C_5_]Inosine was metabolized as extensively as [^13^C_6_]glucose was via glycolysis to produce fully ^13^C-labelled isotopologues of intermediate metabolites, including phosphoenolpyruvate (PEP), pyruvate and lactate. The presence of a substantial fraction of [^13^C_2_]isotopologues among the ^13^C-labelled products of the Krebs cycle (citrate, α-ketoglutarate (αKG), glutamate, glutathione, succinate, fumarate, malate and aspartate) is consistent with the pyruvate dehydrogenase (PDH)-dependent reaction for both inosine- and glucose-tracer treatments (Fig. 2c and Extended Data Fig. 5a). Next, we employed a dual-tracer experiment ([^13^C_5_]inosine and [6,6-D_2_]glucose) to elucidate the carbon allocation of inosine in the presence of glucose. Our results show that active human T cells could simultaneously catabolize inosine-derived ribose and glucose through the PPP, glycolysis and the TCA cycle (Extended Data Fig. 5b and Supplementary Fig. 2). Interestingly, T cells displayed higher oxygen consumption but lower extracellular acidification in inosine-containing conditioned medium than in glucose-containing conditioned medium (Extended Data Fig. 5c). Together, these data support the notion that T_eff_ cells have an extensive capacity to utilize inosine as an alternative to glucose as a fuel source in the central carbon metabolism in vitro.

### Purine nucleoside phosphorylase is required for inosine-dependent proliferation and effector functions in vitro

**Fig. 3:**
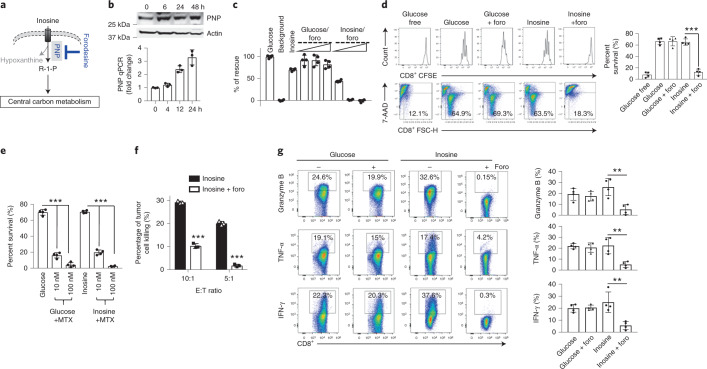
PNP is required for inosine-dependent proliferation and effector functions of mouse T_eff_ cells. **a**, Diagram of the PNP inhibitor foro blocking the breakdown of inosine into hypoxanthine and R1P. **b**, PNP protein and messenger RNA expression levels in mouse T cells at the indicated time points following activation were determined by immunoblot (top) and quantitative PCR (bottom), respectively. Data are representative of two independent experiments. **c**, Bar graph representing mouse T-cell bioenergetic activity in the indicated conditioned media. Activated mouse T cells were incubated without glucose (background), with glucose or with inosine, as well as with glucose or inosine in combination with increased concentrations of foro (0.1 μM, 0.5 μM and 2 μM) for 24 h, followed by Biolog redox dye mix MB incubation, and were measured spectrophotometrically at 590 nm. **d**, Bar graph representing cell-survival percentages in the indicated condidioned media. Naive CD8^+^ cells from C57BL/6 mice were activated as previously described in complete medium for 24 h, and then the cells were switched to the indicated conditioned media in combination with 2 μM foro for 72 h. Cell proliferation and cell death were determined by CFSE dilution (top) and 7-AAD uptake (bottom), respectively. ***P = 0.000009 for inosine versus inosine + foro. **e**, Naive CD8^+^ T cells from C57BL/6 mice were activated and cultured in the presence of glucose or inosine and were treated with MTX for 72 h. Cell death was determined by 7-AAD uptake. ***P = 5.68 × 10^–7^, 1.23 × 10^–7^, 1.07 × 10^–7^ and 3.19 × 10^–10^, from left to right. **f**, B16 melanoma cells were co-cultured with activated Pmel^+^ T cells generated in the presence of inosine with or without foro, and the percentage of tumour-cell lysis was determined by calcein release. ***P = 0.000008 and 0.000007 for E:T ratios 10:1 and 5:1, respectively. **g**, Naive CD8^+^ T cells from C57BL/6 mice were activated as previously described and differentiated in the indicated conditioned media with or without 2 μM foro for 4 d. The indicated proteins were quantified by intracellular staining following PMA and ionomycin stimulation. Data are presented as mean ± s.d. (*n* = 3 for **b**,**f**; *n* = 4 for **c**–**e**,**g**). **P = 0.0042, 0.0048 and 0.0056 for granzyme B, TNF-α and IFN-γ for inosine versus inosine + foro. Data were analysed by unpaired two-sided *t*-test (**d**–**g**). Sample size (*n*) represents biologically independent samples (**b**–**g**). Source data

**Extended Data Fig. 6:**
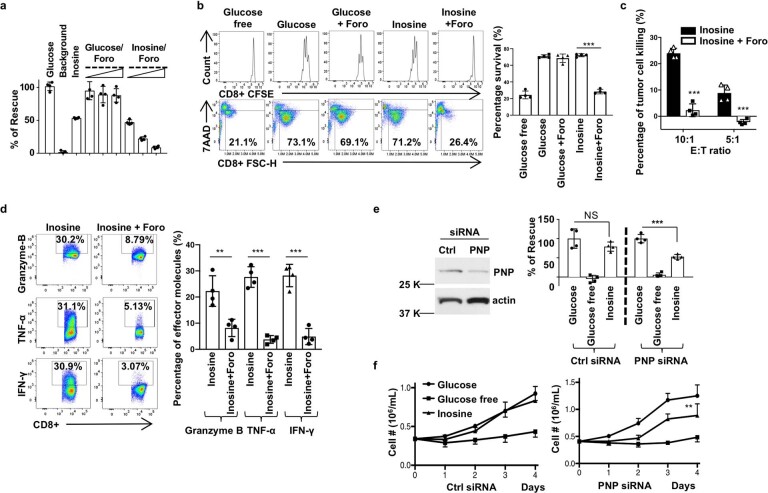
The purine nucleoside phosphorylase (PNP) is required for inosine-dependent human T effector cell proliferation and effector functions. **a**, The same experiment as in Fig. 3c was performed for active human T cells and cell bioenergetic activity was measured. **b**, Naive CD8+ T cells from human PBMCs were activated by plate-bound anti-CD3 and anti-CD28 antibodies in the indicated conditional media with or without 2 μM Foro for 5 days. Cell proliferation and cell death of active CD8+ T cells were determined by CFSE dilution (upper) and 7AAD uptake (lower), respectively. ***, *P*=2E-07 for Inosine versus Inosine+Foro. **c**, GD2-CAR T cells were generated using complete media, then switched to indicated conditional media and cultured for 48-72 h. LAN-1 cells were co-cultured with GD2-CAR T cells in the indicated conditional media, and cell death of LAN-1 was determined by calcein release. ***, *P*=0.000008 and 0.0004 for E:T ratio 10:1 and 5:1 for Inosine versus Inosine+Foro respectively. **d**, The indicated proteins were quantified by intracellular staining following PMA and ionomycin stimulation. **, *P*=0.0058; ***, *P*=0.00003, 0.0001 for Granzyme B, TNF-α, and IFN-γ for Inosine versus Inosine+Foro, respectively. **e**, Human T cells from PBMCs were activated by plate-bound anti-CD3 and anti-CD28 antibodies for 2 days. Activated human T cells were electroporated with scrambled siRNA or PNP siRNA using Neon system and cultured for 2 days. PNP protein expression levels were determined by immunoblot (left). Cells were then incubated without glucose (background), with glucose or with inosine for 24 h, followed by Biolog redox dye mix MB incubation for 2 h and measured spectrophotometrically at 590 nm (right). NS, *P*=0.1865; ***, *P*=0.0003 for Glucose versus Inosine. **f**, Proliferation curves of human T cells transfected with scrambled siRNA (left) or PNP siRNA (right) cultured in the indicated conditional media were determined by counting cell number daily. **, *P*=0.0078 right by Two-Way ANOVA. Data are presented as mean ± SD (n=4 for **a**–**f**). Data were analyzed by unpaired two-sided t-test (**d**, **e**). Sample size (n) represents biologically independent samples (**a**–**f**). Source data

Purine nucleoside phosphorylase (PNP) is responsible for hydrolysing inosine into ribose-1-phosphate, and is substantially upregulated following T-cell activation (Fig. 3a,b)^38^. Given that the ribose subunit of inosine is extensively catabolized in T_eff_ cells, we reasoned that PNP is required for inosine-mediated bioenergetic support. To test this idea, we assessed the bioenergetic activity of T_eff_ cells in the presence of a PNP inhibitor (forodesine (foro); also referred to as BCX-1777, Immucillin H)^41^. As shown in Fig. 3c and Extended Data Fig. 6a, the treatment with the PNP inhibitor foro led to a dosage-dependent attenuation of bioenergetic activity in both mouse and human T_eff_ cells cultured in inosine-containing, but not in glucose-containing, medium. We then assessed the proliferation, viability and effector functions of T_eff_ cells treated with foro. In accordance with its effects on bioenergetic activity, foro significantly dampened proliferation, viability and tumour-killing activity, as well as the expression of effector molecules in mouse (Fig. 3d,f,g) and human (Extended Data Fig. 6b–d) T_eff_ cells cultured in inosine-containing medium. In contrast and as expected, the dihydrofolate reductase (DHFR) inhibitor methotrexate (MTX) that inhibits purine biosynthesis, thymidylate biosynthesis and folate metabolism significantly reduced cell viability in both inosine-containing and glucose-containing medium (Fig. 3e). Next, we sought to validate the data from pharmacological inhibition of PNP by knocking down PNP in human T_eff_ cells. As shown in Extended Data Fig. 6e,f, electroporation with PNP-targeted short interfering RNA (siRNA), but not control scramble siRNA, partially reduced the level of PNP protein in T_eff_ cells, and attenuated the bioenergetic activity and number of human T_eff_ cells cultured in inosine-containing, but not in glucose-containing, medium. Taken together, our results suggest that PNP-dependent inosine hydrolysis is required for inosine-mediated proliferation, bioenergetic support, tumour-killing activity and the effector-molecule expression by T_eff_ cells in vitro.

### PNP inhibition suppresses inosine catabolism in T_eff_ cells in vitro

**Fig. 4:**
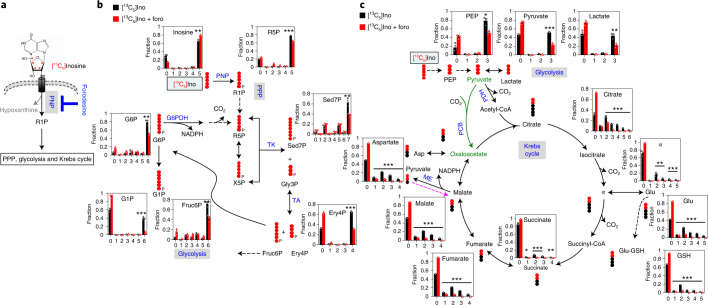
The PNP inhibitor foro suppresses inosine catabolism in T_eff_ cells. **a**, Diagram of the PNP inhibitor foro blocking the catabolism of [^13^C_5_]inosine into R1P and its subsequent entrance into the PPP, glycolysis and the Krebs cycle. The red diamonds denote the uniformly ^13^C-labelled positions of the ribose subunit of inosine. **b**,**c**, Active human T cells were incubated in [^13^C_5_]inosine-containing medium with or without foro for 24 h, extracted and analysed. as described in the Methods. for PPP metabolites (**b**) and glycolytic and Krebs cycle metabolites (**c**) by IC–UHR-FTMS and by 1D HSQC NMR for inosine. All symbols and abbreviations are as described in Fig. 2. Numbers on the *x* axes represent the numbers of ^13^C atoms in the given metabolites. Values represent mean ± s.e.m. (*n* = 3). *, **, and *** denote *P* < 0.05, 0.01 and 0.001, respectively. In **b**, **P = 0.0012, 0.0034, 0.0043 and 0.0064 for [^13^C_5_]inosine, [^13^C_6_]G6P, [^13^C_6_]Fruc6P and [^13^C_7_]Sed7P for [^13^C_5_]inosine versus [^13^C_5_]inosine + foro, respectively; ***P = 0.00096, 0.0002 and 0.000005 for [^13^C_5_]R5P, [^13^C_6_]G1P and [^13^C_4_]Ery4P for [^13^C_5_]inosine versus [^13^C_5_]inosine + foro, respectively. In **c**, *P = 0.0117; ***P = 0.0002; **P = 0.0087 for [^13^C_3_]PEP, [^13^C_3_]pyruvate and [^13^C_3_]lactate for [^13^C5]inosine versus [^13^C5]inosine + foro, respectively. Other *P* values are listed in Supplementary Table 2. Data were analysed by unpaired two-sided *t*-test (**b**,**c**). Sample size (*n*) represents biologically independent samples (**b**,**c**). Source data

**Extended Data Fig. 7:**
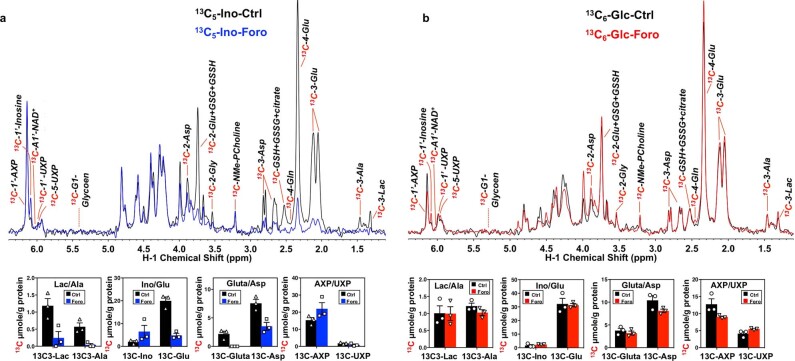
PNP inhibitor forodesine blocks ^13^C incorporation from [^13^C_5_]-ribose-inosine into central metabolites but has little effect on [^13^C_6_]-glucose catabolism. The same experiment as in Fig. 4 was performed using active human T cells with or without Foro in the presence of [^13^C_5_]-Ino or [^13^C_6_]-Glc for 24 h. The polar extracts from both tracer experiments were analyzed by 1D HSQC NMR for ^13^C abundance (spectra as shown). Selected metabolites were quantified as ^13^C μmole/g protein using the N-methyl resonance of phosphocholine (NMe-PCholine) as an internal standard, which are shown as bar graphs for the [^13^C_5_]-Ino (**a**) and [^13^C_6_]-glucose tracer (**b**) experiments. Values are average ± SEM (n=3). Sample size (n) represents biologically independent samples. Dashed line denotes the expected peak position of the H1-glucose resonance of glycogen. The proton resonances used for quantification were: ^13^C_3_-Lac/Ala – the H3 resonances of lactate/Ala; ^13^C-Gluta – the H4-Glu resonance of GSH + GSSG with minor contribution of H2,5 resonance of citrate; ^13^C-Ino, ^13^C-Glu, ^13^C-Asp, ^13^C-AXP, and ^13^C-UXP – H1’, H4, H3, and H1’ resonances of inosine, glutamate, aspartate, and adenine/uracil nucleotides, respectively. Source data

Next, we assessed the effect of PNP inhibition on inosine catabolism by comparing the carbon utilization of [^13^C_5_]inosine in the presence and absence of the PNP inhibitor (foro) in T_eff_ cells (Fig. 4a). Because foro treatment significantly reduces T-cell viability in inosine-containing medium (Fig. 3d and Extended Data Fig. 6b), we chose to moderately suppress PNP activity by applying a lower dose of foro and used a shorter incubation time than in the previous experiment. Treatment with foro led to the fractional enrichment of [^13^C_5_]inosine, the metabolic substrate of PNP, and a decrease in the fractional enrichment of the fully ^13^C-labelled species of PPP metabolites, which are indirect metabolic products of PNP (Fig. 4b). These results suggest that the inhibition of PNP suppresses the catabolism of the ribose subunit of inosine via the PPP. Next, we tracked the fate of [^13^C_5_]inosine via glycolysis and the Krebs cycle in the context of suppressed PNP activity. As shown by IC–UHR-FTMS analysis (Fig. 4c), the foro treatment greatly reduced the fraction of fully ^13^C-labelled isotopologues of glycolysis metabolites and the fraction of [^13^C_2_]isotopologues of Krebs-cycle metabolites. To further assess the effect of foro on T-cell metabolism both in glucose- and in inosine-containing media, we applied one-dimensional (1D) (^1^H,^13^C)-heteronuclear single-quantum correlation nuclear magnetic resonance ((^1^H,^13^C)-HSQC NMR) and 1D ^1^H NMR to quantify ^13^C-labelled isotopologues of several representative metabolites of glycolysis, the Krebs cycle and nucleotide biosynthesis, including lactate, alanine, inosine, glutamate, glutathione and oxidized glutathione, aspartate, adenine nucleotides and uracil nucleotides (Extended Data Fig. 7). Foro treatment substantially altered the quantity of [^13^C_5_]inosine-derived metabolites (Extended Data Fig. 7a). In contrast, foro had little effect on the quantity of [^13^C_6_]glucose-derived metabolites (Extended Data Fig. 7b). Together, these data suggest that T_eff_ cells have an extensive capacity for hydrolysing inosine as an alternative fuel source to glucose in the central carbon metabolism in vitro.

### Transformed cells display a diverse capacity for utilizing inosine as a carbon source

**Fig. 5:**
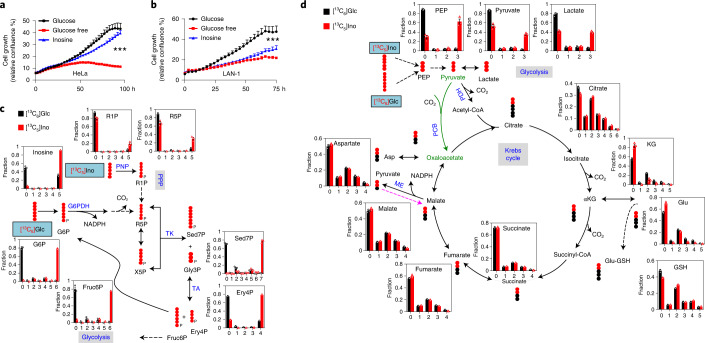
Transformed cells display a diverse capacity to utilize inosine as a carbon source. **a**, Growth curves for HeLa cells in the indicated conditioned media were determined by live-cell imaging analysis (IncuCyte ZOOM). Data are presented as mean ± s.d. (*n* = 4). ***P < 0.0001 for 2 mM inosine versus glucose free by two-way analysis of variance (ANOVA). Data are representative of three independent experiments. **b**, LAN-1 cells were cultured in the indicated conditioned media, and cell-growth curves were monitored and analysed by IncuCyte ZOOM. Data are presented as mean ± s.d. (*n* = 4). ***P < 0.0001 for 2 mM glucose versus 2 mM inosine by two-way ANOVA. Data are representative of three independent experiments. **c**,**d**, HeLa cells were incubated with [^13^C_6_]glucose or [^13^C_5_]inosine for 24 h, extracted as described in the Methods and analysed for PPP metabolites (**c**) and glycolytic and Krebs-cycle metabolites (**d**) by IC–UHR-FTMS. All symbols and abbreviations are as described in Fig. 2. Numbers on the *x* axes represent the numbers of ^13^C atoms in given metabolites. Values represent mean ± s.e.m. (*n* = 3). Sample size (*n*) represents biologically independent samples (**a**–**d**). Source data

**Extended Data Fig. 8:**
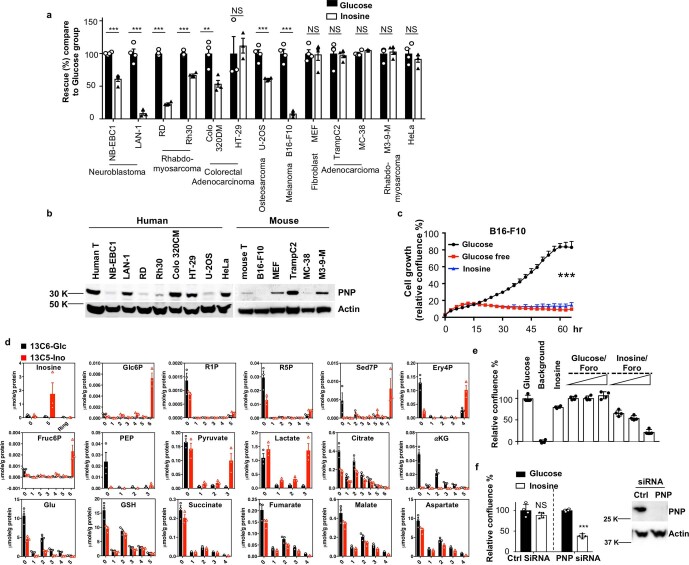
Transformed cells display diverse capacity to utilize inosine as a carbon source. **a**, The indicated cell lines were cultured without glucose (background), with glucose or with inosine, and cell growth curves were monitored by live cell imaging analysis (IncuCyte ZOOM™). Cell confluences of the indicated cell lines cultured with glucose at 72-96 h were set to 100%. Data are presented as mean ± SD (n=4). **, *P*<0.01 and ***, *P*<0.001 for Glucose versus Inosine. *P=*0.00009, 0.0001, 0.0000001, 0.00001, 0.0042, 0.6917, 0.0006, 0.00001, 0.9059, 0.7962, 0.0796, 0.6663, 0.3829 from left to right, respectively. **b**, The expressions of PNP in indicated cell lines were determined by immunoblot. **c**, B16-F10 cells were cultured in the indicated conditional media and cell growth curves were monitored and analyzed by IncuCyte ZOOM™. Data are presented as mean ± SD (n=4). ***, *P*<0.001 for 2 mM Glucose versus 2 mM Inosine. Data are representative of three independent experiments. **d**, Quantification of indicated metabolites in HeLa cells. All abbreviations are as described in Fig. 2. Values represent mean ± SEM (n=3). **e**, HeLa cells were incubated without glucose (background), with glucose or with inosine, as well as in combination with increased concentrations of Foro (0.1 μM, 0.5 μM, and 2 μM), and cell growth was monitored by live cell imaging analysis (IncuCyte ZOOM™). The confluences of HeLa cells cultured with glucose at 60 h were set to 100%. Data are presented as mean ± SD (n=4). **f**, HeLa cells were transfected with scrambled siRNA or PNP siRNA for 3 days. Cells were then switched to glucose-free (background), glucose-containing, or inosine-containing media and cell confluence was determined by live cell imaging analysis (IncuCyte ZOOM™). The confluences of HeLa cells in the presence of glucose at 96 h were set to 100%. Error bars represent standard deviation from mean of quadruplicate samples (left). NS, *P=*0.2022; ***, *P=*0.0000006 for Glucose versus Inosine. PNP protein expression levels were determined by immunoblot (right). Data are representative of two independent experiments (**b**, **e**, **f**). Data were analyzed by unpaired two-sided t-test (**a**, **c**, **f**). Sample size (n) represents biologically independent samples (**a**–**f**). Source data

The nucleoside transporters and PNP are expressed in the majority of tissues and cell lines (data from The Human Protein Atlas). We therefore reasoned that cancer cells may be able to take up and utilize inosine as an alternative carbon source. To this end, we selected a panel of transformed cell lines and compared their growth rate in glucose- versus inosine-supplemented glucose-free medium. Although of the tested cell lines displayed some degree of inosine-dependent proliferation, several cell lines were unable to grow in inosine-containing medium (Fig. 5a,b and Extended Data Fig. 8a,c). We examined the level of PNP in these cell lines and found that it could not predict each cell line’s capability of utilizing inosine to support cell growth (Extended Data Fig. 8b). Next, we chose HeLa as a model cell line for our study of inosine catabolism and the role of PNP in inosine-dependent growth. Applying the same metabolic approach (Fig. 2a) that we used to study T cells, we compared glucose and inosine catabolism in HeLa cells. The pattern of ^13^C incorporation into intermediate metabolites in the central carbon metabolic pathways that HeLa cells displayed was similar to that of T cells. Collectively, HeLa cells extensively catabolized [^13^C_6_]glucose and [^13^C_5_]inosine, which derived fully ^13^C-labelled isotopologues of intermediate metabolites in the PPP and glycolysis, as well as a substantial fraction of [^13^C_2_]isotopologues in the Krebs cycle metabolites (Fig. 5c,d and Extended Data Fig. 8d). Consistent with the indispensable role of PNP in inosine catabolism in T cells, the pharmacological inhibition of PNP via foro, or genetic knockdown of PNP via siRNA, significantly dampened inosine-dependent bioenergetics activity and cell growth in HeLa cells (Extended Data Fig. 8e,f). Together, these data suggest that some cancer cells are capable of utilizing inosine as an alternative fuel source instead of glucose in central carbon metabolism.

### Inosine enhances the efficacy of T-cell-based immunotherapy in solid tumours

**Fig. 6:**
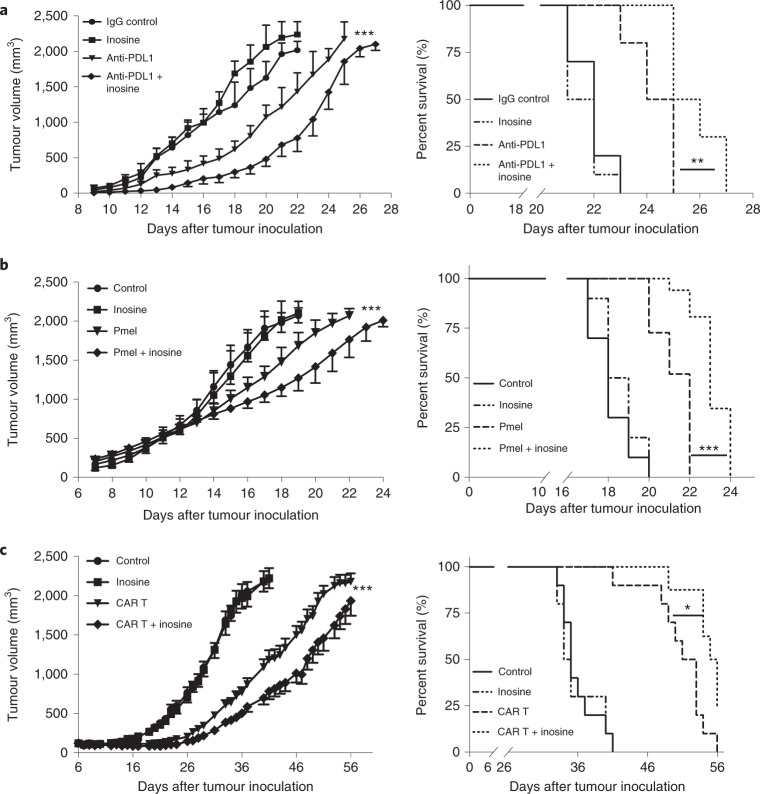
Inosine supplementation enhances immunotherapy in targeting solid tumours that are defective in metabolizing inosine. **a**, A murine melanoma xenograft model was established in C57BL/6 mice by subcutaneous inoculation of B16-F10 tumour cells. The indicated experimental mice were treated with IgG control (200 μg, intraperitoneal (i.p.) injection twice per week), inosine (300 mg per kg (body weight), oral gavage daily), anti-PDL1 antibody (200 μg, i.p. twice per week) or anti-PDL1 antibody (200 μg, i.p. twice per week) + inosine (300 mg per kg (body weight), oral gavage daily). Tumour size and mouse survival were monitored. Data represent mean ± s.d. (*n* = 10). ***P < 0.0001 for anti-PDL1 versus anti-PDL1 + inosine, by two-way ANOVA (left). **P = 0.0018 for anti-PDL1 versus anti-PDL1 + inosine (right), by one-sided Mantel–Cox test. **b**, C57BL/6 mice were injected (subcutaneously) with B16-F10 melanoma cells and were sublethally irradiated (500 cGy) at day 6 after tumour-cell inoculation. One day later, mice were i.v. injected with activated Pmel CD8^+^ T cells (4 × 10^6^ cells per mouse). Mice were administered inosine (300 mg per kg (body weight) per day by oral gavage from day 8). Tumour size and mouse survival were monitored. Data are presented as mean ± s.d. (*n* = 10). ***P = 0.0091 for Pmel versus Pmel + inosine, by two-way ANOVA (left). **P < 0.0001 for Pmel versus Pmel + inosine with one-sided Mantel–Cox test (right). **c**, A human neuroblastoma xenograft model was established in NSG mice by subcutaneous inoculation of LAN-1 neuroblastoma cells. The indicated experimental mice were treated, from day 6 when tumours reached 100–150 mm^3^, with PBS (i.v. as control), inosine (300 mg per kg (body weight) by oral gavage daily), GD2-CAR T cells (8 × 10^6^ cells per mouse, i.v.) and GD2-CAR T cells (8 × 10^6^ cells per mouse, i.v.) + inosine (300 mg per kg (body weight) by oral gavage daily). Tumour growth and mouse survival were monitored. Data are presented as mean ± s.e.m. (*n* = 20 for control and inosine, 19 for CAR T and 16 for CAR T + inosine group). ***P < 0.0001 for CAR T versus CAR T + inosine with two-way ANOVA (left). *P = 0.0111 for CAR T versus CAR T + inosine with one-sided Mantel–Cox test (right). Data are representative of two independent experiments (**a**–**c**). Sample size (*n*) represents biologically independent animals (**a**,**b**) or tumours (**c**). Source data

**Extended Data Fig. 9:**
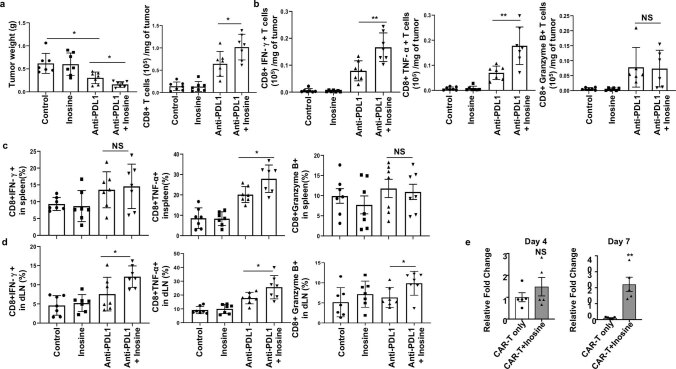
Inosine supplement enhances T cells infiltration and effector molecule expression. **a**, Murine melanoma xenograft model was established in C57BL/6 mice by subcutaneous inoculation of B16-F10 tumor cells. After 15 days of indicated treatment, mice were euthanized and tumor mass was harvested and weighed and the absolute number of tumor infiltrated T_eff_ cells were calculated. *, *P*=0.0112 and 0.0127 for control versus anti-PDL1 and anti-PDL1 versus anti-PDL1+Inosine (left); *, *P*=0.0295 for anti-PDL1 versus anti-PDL1+Inosine (right). (**b**–**d**) At day 15, tumors, spleen and draining lymph nodes (dLN) with indicated treatments were collected and the levels of intracellular effector molecules were determined by FACS; Values represent mean ± SD (n=7). **b**, **, *P*=0.0038, 0.0037; NS, *P*=0.9079 for anti-PDL1 versus anti-PDL1+Inosine from left to right. (**c**) NS *P*=0.7557; *, *P*=0.0210; NS, *P*=0.7778 for anti-PDL1 versus anti-PDL1+ inosine from left to right. **d**, *, *P*=0.0391, 0.0476, 0.0326 for anti-PDL1 versus anti-PDL1+Inosine from left to right. **e**, Human neuroblastoma xenograft model was established in NSG mice by subcutaneous inoculation of LAN-1 neuroblastoma cells, followed by i.v. administration of GD2-CAR-T-Luciferase cells only or in combination with oral gavage of inosine (300 mg/kg/day) when tumors grew to 4-6 mm in diameter. T cell infiltration in tumor was monitored at indicated time by IVIS imaging. Data are presented as mean ± SEM (n=5). NS P=0.3355; **, *P*=0.0015 for CAR-T only versus CAR-T + inosine. Data are representative of two independent experiments. Sample size (n) represents biologically independent samples (**a**–**d**) and animals (**e**). Data were analyzed by unpaired two-sided t-test (**a**–**e**). Source data

T_eff_ cells represent a key component of anti-tumour immunity. The checkpoint-blockade approach, which involves monoclonal antibodies targeting PD-1/PDL1 and CTLA4, and adoptive cell transfer of tumour-infiltrating lymphocytes (TILs) or CAR T cells are two front-line T-cell-based immunotherapies^21,23^. On the basis of the in vitro findings described above, we reasoned that supplementation with inosine may improve T_eff_-cell-mediated anti-tumour activity, particularly towards tumour cells such as LAN-1 (Fig. 5b) and B16-F10 (Extended Data Fig. 8c), which are unable to utilize inosine to support cell growth. Blockade of the PDL1 and PD-1 (PD) pathway has been shown to elicit durable T-cell-dependent anti-tumour responses. We thus assessed the anti-tumour effect of a combination of inosine and anti-PDL1 antibody in B16-melanoma-bearing mice. Although tumour growth, the number of tumour-infiltrating T cells and animal survival were comparable between mice treated with vehicle (IgG control) and those treated with inosine, the monotherapy using the anti-PDL1 antibody clearly delayed tumour growth, and prolonged animal survival time (Fig. 6a and Extended Data Fig. 9a). Importantly, mice treated with the combination of inosine and anti-PDL1 antibody displayed a better outcome and had more tumour-infiltrating T cells than did the monotherapy group (Fig. 6a and Extended Data Fig. 9a). In addition, the combination of inosine supplementation and anti-PDL1 treatment increased the number of CD8^+^ TILs expressing IFN-γ and TNF-α compared with anti-PDL1 treatment alone (Extended Data Fig. 9b). Similarly, the combined treatment moderately increased the percentage of circulating CD8^+^ T cells that expressed IFN-γ in draining lymph nodes, and of those that expressed TNF-α in both spleen and draining lymph nodes (Extended Data Fig. 9c,d). We also examined the ability of inosine to potentiate adoptive-transfer-based therapy, which transfers Pmel^+^ T cells to mice bearing B16-F10 xenografts (Fig. 6b) or GD2-CAR T cells to immune-deficient mice bearing GD2-positive human neuroblastoma (LAN-1) xenografts (Fig. 6c). Adoptively transferred T cells alone significantly delayed tumour growth and prolonged survival of the animals, and treatment with inosine plus T cells further potentiated these effects in the mouse xenograft models (Fig. 6b,c and Extended Data Fig. 9e). Taken together, our studies suggest that inosine supplementation enhances the potency and durability of T-cell-based cancer immunotherapy in mouse preclinical models.

**Extended Data Fig. 10:**
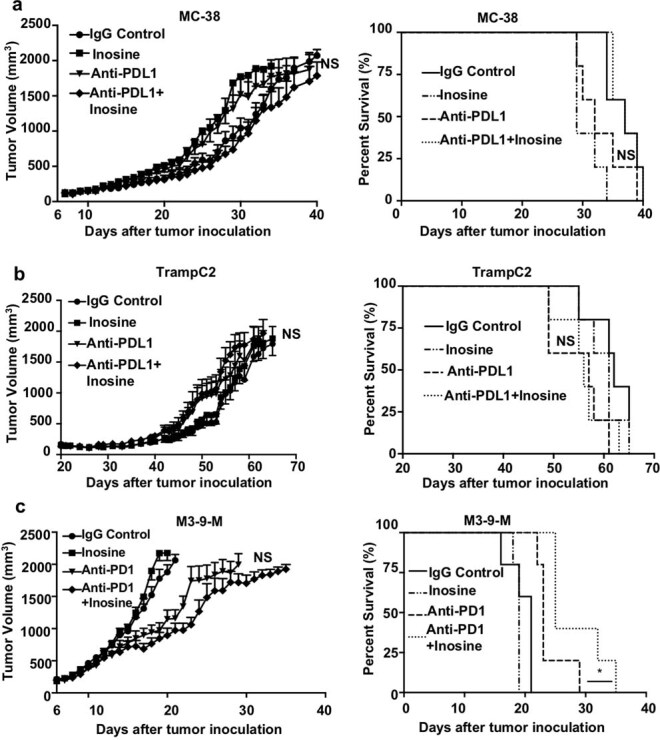
Inosine supplement fails to enhance immunotherapy in MC-38, TrampC2 and M3-9-M tumor models. (**a**–**c**) Murine xenograft model was established in C57BL/6 mice by subcutaneous inoculation of indicated tumor cells. The indicated experimental mice were treated with IgG control (200 μg, i.p. twice/week), inosine (300 mg/kg, oral gavage every day), anti-PDL1 or anti-PD1 antibody (200 μg, i.p. twice/week), and anti-PDL1 or anti-PD1 antibody (200 μg, i.p. twice/week) + inosine (300 mg/kg, oral gavage daily). Tumor size and mouse survival were monitored. Data are presented as mean ± SEM (n=7, 9, 9, 10 for IgG Control, Inosine, Anti-PDL1, Anti-PDL1+Inosine for MC-38; n=7, 10, 9, 10 for IgG Control, Inosine, Anti-PDL1, Anti-PDL1+Inosine for TrampC2; n= 8, 9, 9, 9 for IgG Control, Inosine, Anti-PD1, Anti-PD1+Inosine for M3-9-M). Sample size (n) represents biologically independent tumors. Tumor progression curves (left) were analyzed with Two-Way ANOVA and mouse survival curves (right) were analyzed with one-sided Mantel-Cox test. NS, *P*=0.4544, 0.9999, 0.3601 for antibody versus antibody+inosine for tumor progression curves of MC-38, TrampC2, and M3-9-M, respectively. NS, *P*=0.1255, 0.8822; *, *P*=0.0390 for antibody versus antibody+inosine for survival curves of MC-38, TrampC2, and M3-9-M, respectively. Data are representative of two independent experiments (**a**–**c**). Source data

Next, we sought to determine whether inosine supplementation could still enhance the T-cell-mediated anti-tumour response while the tumour cell lines can take up and catabolize inosine. To answer this question, we chose MC-38, TrampC2 and M3–9-M cell lines, which are all capable of using inosine as an alternative energy source in the absence of glucose (Extended Data Fig. 8a). In all three models, inosine supplementation combined with immune checkpoint blockade (anti-PDL1 or anti-PD-1 antibody) failed to provide a statistically significant benefit in comparison with antibody treatment alone, except for the marginal improvement in mouse survival in the M3–9-M xenograft model (Extended Data Fig. 10a–c). These data suggest that the beneficial effects of inosine supplementation are diminished when tumour cells are able to compete with T cells for utilization of inosine.

## Discussion

T cells are distributed throughout the body and thus encounter variable metabolic stresses depending on where tumours or infections occur. A key factor affecting T-cell functionality could be the competition for nutrients between infiltrating T cells and rapidly proliferating cells, including cancer cells or pathogens. It is likely that a wide range of abundant bioenergetic carbohydrates in blood plasma could be a source of nutrients for proliferative T cells and cancer cells in vivo^42^. Mounting evidence suggests that cancer cells can scavenge extracellular protein or utilize acetate and ketone bodies as alternative fuels^43,44^. Although glucose is considered to be the primary fuel for proliferating T cells, galactose can replace glucose to support T-cell survival and proliferation following activation^45^. Our studies have revealed the capacity of T cells to utilize inosine as an energy source to support effector-cell functions in vitro. Such metabolic plasticity is likely to be crucial for the ability of T cells to elicit robust immune responses in different tissue contexts. However, this needs to be further tested by in vivo metabolomics experiments, because T cell in vitro carbon usage (such as glucose usage) can be different from carbon usage in vivo^46^.

The expression of PNP that can convert inosine into R1P and hypoxanthine is consistent with our findings on inosine’s capacity as a ribose donor in T cells. Such PNP-mediated breakdown of inosine is energetically more efficient than free ribose metabolism, as it only requires inorganic phosphate, whereas the latter must have ATP. We found that the ribose derived from inosine was catabolized efficiently through the central carbon metabolic routes, including the PPP, glycolysis and the Krebs cycle, in place of glucose. This, together with inosine’s ability to support T_eff_-cell growth and function in the absence of glucose, suggests that inosine, as a ribose donor, may replace glucose as a key fuel source for T_eff_ cells under glucose deprivation, as is found in solid tumours. In addition to the function of inosine-derived ribose as an alternative carbon source for T cell catabolism, we envision that additional mechanisms are involved in mediating some inosine-dependent effects. The ribose subunit of inosine is metabolized more efficiently through the non-oxidative branch of the PPP and glycolysis to produce more PEP than in glucose catabolism. Higher production of PEP in the inosine than in the glucose group could also be attributed to higher activity of glycolytic enzymes, including GAPDH, in the inosine group. These results are consistent with a role for GAPDH and the signalling metabolite PEP in regulating the effector function of T cells^30,45^. Also, inosine-derived hypoxanthine can be converted into uric acid, which may provide additional beneficial effects on T cells as previous studies suggested^39,40^.

The degradation of nucleotides and nucleosides is an evolutionarily conserved metabolic process that can supply carbon and energy to support cell proliferation and function^47–49^. However, we found that inosine, not other nucleosides (adenosine and guanosine), supported T-cell proliferation. This result suggests that inosine is a preferred substrate of nucleoside transporters and nucleoside phosphorylases that are predominantly expressed in T cells. Furthermore, a PNP inhibitor abolished inosine uptake and metabolism as well as the ability to support T_eff_-cell proliferation and functions in vitro. Several previous studies have demonstrated inosine’s role in supporting bioenergetic activity and function in human red blood cells and in swine and chicken erythrocytes, which lack glucose transporters, as well as its ability to enhance the recovery of ischaemic heart and kidney by preserving ATP^50,51^.

Inosine and adenosine can maintain ATP during hypoxia and nutrient restriction in the central nervous system (CNS), and the catabolism of the ribose subunit of inosine is necessary to provide bioenergetic support for CNS cells. The protective effect of adenosine depends on adenosine deaminase (ADA), which converts adenosine to inosine^52^ . Although inosine and adenosine concentrations in blood plasma are in the low micromolar range, they can accumulate to hundreds of micromolar via catabolism of nucleotides and nucleosides in the necrotic, hypoxic or inflammatory microenvironment, which is characteristic of solid tumours^53,54^. T cells may utilize inosine and adenosine accumulated in the TME as an important carbon and energy source. In addition, T cells are capable of utilizing acetate as alternative substrate to support bioenergetic activity and effector function in the TME^55^.

In response to tissue injury, stressed or damaged cells release ATP and its product, adenosine, into the extracellular space to elicit autocrine- and paracrine-signalling responses through cell-surface P2 purinergic receptors^36^. To terminate adenosine-mediated signalling, ADA converts adenosine to inosine, rendering inosine a weak agonist of adenosine receptor^56^. Consistent with these findings, we found that inosine, but not adenosine, enhances T_eff_-cell functions, which may be mediated via inosine catabolism, as the PNP inhibitor abolished both inosine catabolism and inosine-induced activation of T_eff_-cell functions. However, it is still conceivable that some of the in vivo effects of inosine are mediated by purinergic signalling, owing to the concurrent depletion of immune-suppressive adenosine. We therefore surmise that ADA treatment may reduce immunosuppressive adenosine in TME while increasing the levels of inosine to fuel tumour infiltrated T cells, thereby improving anti-tumour immunity. The genetic disorder leading to the loss of ADA enzymatic function can cause severe combined immunodeficiency (SCID)^57^. As such, the polyethylene-glycol-conjugated adenosine deaminase (PEG–ADA) and ADA gene therapy have been developed as the first enzyme-replacement therapy to treat ADA-SCID^58^. On the basis of our findings, further research on the molecular action of PEG–ADA and its repurposing in cancer immunotherapy is warranted.

Anti-tumour immunotherapy not only offers the potential of high selectivity in targeting and killing tumour cells, but also represents an appealing addition to the current therapeutic regimens because of its potential to eradicate recurrent/metastatic disease following conventional therapies. However, the immunosuppressive microenvironment is a major barrier to the development of effective immunotherapy. It has been shown that metabolic modulation of T cells is a valid approach to altering T-cell-mediated immune responses in a variety of physio-pathological contexts^9,10,29,30,33,59–61^. Clearly, nutrient restriction in solid tumour can have an important role in restraining the anti-tumour activity of infiltrating T cells. We have shown that inosine readily replaced glucose in supporting T_eff_-cell growth and function in vitro, and supplementation with inosine improved T_eff_-cell-mediated anti-tumour activities in animal models. Inosine supplementation via oral administration or intravenous (i.v.) infusion has been shown to be safe and tolerable in recent clinical trials for treatment of multiple sclerosis and Parkinson’s disease^62^. We therefore envision that metabolic modulation of T cells, including through inosine and PEG–ADA supplementation, poses a new complementary strategy to optimize the potency and durability of cancer immunotherapy. Additional studies on the formulation of inosine supply or on strategies that may enhance the local accumulation of inosine in the TME are warranted to facilitate future clinical development of metabolic modulation in cancer immunotherapy.

## Methods

### Culture of tumour cells and reagents

B16-F10, HeLa, Colo 320DM, Tramp-C2, MC-38, HT-29 and U-2OS cell lines were acquired from American Type Culture Collection (ATCC). Human neuroblastoma cell line NB-EBC1 and human rhabdomyosarcoma cell lines RD and Rh30 were kindly provided by P. Houghton. LAN-1, a GD2-positive tumour cell line, was a gift from X. Song. M3–9-M cells were kindly provided by C. Mackall^63^. Modified B16-gp100 melanoma cells were kindly provided by N. Restifo^64^. Immortalized mouse embryonic fibroblasts (MEF) cells were generated with C57BL/6 mouse E12.5–14.5 embryos^65^. HT-29 and U-2OS cell lines were cultured in McCoy’s 5A Modified Medium (Gibco) with 10% foetal calf serum (Gibco) and 1% penicillin–streptomycin (Corning) in a 37 °C humidified atmosphere of 95% air and 5% CO_2_. Colo 320DM, RD, Rh30, LAN-1 and M3–9-M cells were grown in RPMI 1640 medium (Corning) with 10% foetal calf serum and 1% penicillin–streptomycin in a 37 °C humidified atmosphere of 95% air and 5% CO_2_. B16-F10, HeLa, NB-EBC1 and MEF cells were grown at 37 °C/5% CO_2_ in DMEM (Corning) supplemented with 10% foetal calf serum and 1% penicillin–streptomycin. Glucose-free DMEM (Sigma-Aldrich) was supplemented with 10% (vol/vol) heat-inactivated dialysed FBS, which was made by dialysis against 100 volumes of PBS (5 changes in 3 d) using Slide-ALyzer G_2_ dialysis cassettes with a 2K molecular-weight cutoff (Thermo Fisher Scientific) at 4 °C. All the chemicals and antibodies used are listed in Supplementary Table 1 and the Reporting Summary.

### Isolation and culture of human and mouse T cells

PBMCs were collected from healthy donors, approved by Baylor College of Medicine review board. Mononuclear cells from PBMCs were isolated by Ficoll/Hypaque density gradient centrifugation. In some experiments, human CD3^+^ T cells were directly enriched from PBMCs by negative selection using MojoSort Human CD3 T Cell Isolation Kit (Biolegend) following the manufacturer’s instructions. Isolated human mononuclear cells or enriched human T cells were either maintained in culture medium containing 10 ng ml^–1^ human IL-7 or were stimulated with plate-bound anti-CD3 and anti-CD28 antibodies in medium containing human IL-2 (100 U ml^–1^). Plates were pre-coated with 1 μg ml^–1^ anti-CD3 and 1 μg ml^–1^ anti-CD28 antibodies overnight at 4 °C. To generate GD2-CAR T cells, activated human T cells were transduced with GD2-CAR retrovirus on RetroNectin-coated non-tissue-culture plates and were cultured with IL-2 for up to 1 week (ref. ^66^).

Mouse T cells were enriched from spleens and lymph nodes by negative selection using a magnetic-activated cell sorting (MACS) system (Miltenyi Biotec) or MojoSort mouse CD3/CD8 T Cell Isolation Kit (Biolegend) following the manufacturer’s instructions. For total-T-cell culture, freshly isolated total mouse T cells with 75–80% CD3 positivity were either maintained in culture medium containing 5 ng ml^–1^ mouse IL-7 or stimulated with mouse IL-2 (100 U ml^–1^) as well as plate-bound anti-mouse CD3 (clone 145-2C11) and anti-mouse CD28 (clone 37.51) antibodies. Plates were pre-coated with 2 μg ml^–1^ anti-CD3 and 2 μg ml^–1^ anti-CD28 antibodies overnight at 4 °C. For mouse and human T-cell CFSE dilution analysis, 1 x 10^7^–2 x 10^7^ cells were pre-incubated for 10 min in 4 μM CFSE (Invitrogen) diluted in PBS plus 5% FBS before culture. For CD8^+^-T-cell activation and culture, naive CD8 T cells were activated by plate-bound antibodies and incubated with recombinant mouse IL-2 (100 U ml^–1^) and IL-12 (5 ng ml^–1^) for 5 d. For Pmel^+^CD8^+^-T-cell activation and culture, splenocytes from Pmel transgenic mice were isolated and cultured with 1 μM human gp100 (hgp100) (GenScript) and 30 U ml^–1^ recombinant human IL-2 (PeproTech) in complete medium for 5 d^67,68^. The cells were then cultured in RPMI 1640 medium supplemented with 10% (vol/vol) heat-inactivated FBS, 2 mM l-glutamine, 0.05 mM 2-mercaptoethanol, 100 U ml^–1^ penicillin and 100 μg ml^–1^ streptomycin at 37 °C in 5% CO_2_. Glucose-free RPMI 1640 medium (Gibco) supplemented with 10% (vol/vol) heat-inactivated dialysed FBS (DFBS) was used as basal conditioned medium for glucose and inosine reconstitution. DFBS was made by dialysis against 100 volumes of PBS (5 changes in 3 d) using Slide-ALyzer G2 dialysis cassettes with a 2K molecular-weight cutoff (Thermo Fisher Scientific) at 4 °C.

### Flow cytometry

For the analysis of surface markers, cells were stained in PBS containing 2% (wt/vol) BSA and the appropriate antibodies. For intracellular cytokine staining, 5 x 10^5^ naive CD8^+^ T cells were activated and cultured in 0.5 ml complete RPMI medium for 3–4 d, followed by restimulation by direct addition of stimulation cocktails of PMA, ionomycin, brefeldin A and monensin (500×) into the medium and then were incubated for 4 h. After incubation, cells were washed twice with PBS and resuspended in PBS containing 2% (wt/vol) BSA. Cells were then fixed and permeabilized using Foxp3 Fixation/Permeabilization solution according to the manufacturer’s instructions (eBioscience). Cells were then stained with the indicated antibodies listed in Supplementary Table 1 and the Reporting Summary. Flow cytometry data were acquired on Novocyte (ACEA Biosciences) or LSRII (Becton Dickinson) and were analysed with FlowJo software (TreeStar), and gating strategies are shown in Supplementary Fig. 3.

### Metabolite-screening assay

Metabolite screening was performed using 96-well PM-M1 and PM-M2 phenotyping microarray for mammalian cells (BioLog). Human or mouse activated T cells were washed twice with PBS, and were resuspended in glucose and phenol-red-free DMEM (Sigma-Aldrich) supplemented with 10% dialysed FBS and 100 U ml^–1^ IL-2 followed by immediate inoculation of 100 μl cells per well (1 × 10^6^ cells ml^–1^) into the Biolog plates. After 24 h of incubation at 37 °C in a 5% CO_2_ incubator, 20 μl of Redox Dye Mix MB (BioLog) was added to each well, and absorbance at 590 nm and 750 nm wavelengths were recorded at different time points^69^.

### Cytotoxicity assay

The target tumour cells (B16-gp100 mouse melanoma or LAN-1 human neuroblastoma cells) were stained with calcein for determining cytotoxic potential of Pmel^+^ T_eff_ or GD2-CAR T cells by the calcein release assay^70^. For staining target cells, a 2 μg ml^–1^ Calcein AM (Invitrogen) staining medium was prepared (1 mg ml^–1^ stock) in complete RPMI medium. The tumour cells were resuspended at 1 × 10^6^ cells ml^–1^ in Calcein AM-staining medium and were incubated for 30 min at 37 °C in a 5% CO_2_ incubator with intermittent mixing. The target cells were washed and resuspended at 1 × 10^5^ cells ml^–1^ in RPMI complete medium. Pmel^+^ T_eff_ or GD2-CAR T cells pre-cultured in conditioned medium for 48–72 h were resuspended at 1 × 10^6^ cells ml^–1^ in RPMI complete medium, and three serial dilutions was performed. Aliquots of 100 μl from each CAR T cell serial dilution containing 1 × 10^5^, 0.5 × 10^5^ and 0.25 × 10^5^ cells were added per well in a 96-well U-bottom plate, in quadruplicates. Aliquots of 100 μl of calcein-loaded tumour cells were added (1 × 10^4^ cells per well) to each of these wells to generate E:T ratios of 10:1, 5:1 and 2.5:1. Maximum and spontaneous release controls were set up in 6 replicates using 1% Triton X-100 (final concentration) and plain medium, respectively. The plate was spun at 100*g* for 2 min and incubated for 4 h at 37 °C in a 5% CO_2_ incubator. After the 4-h incubation, the cells were gently mixed to evenly distribute the released calcein in the supernatant, and the plate was spun at 400*g* for 3 min to pellet the cells and any debris. Then, 100 μl supernatant was recovered and transferred to a flat-bottom plate. The fluorescence was read using a Spectramax M2 microplate reader (excitation, 485 nm; emission, 528 nm). The percent specific lysis was calculated using the formula ((test release − spontaneous release) / (average maximum release − average spontaneous release)) x 100.

### Mice

C57BL/6NHsd mice were purchased from Envigo. NSG mice (NOD-scid IL2Rgamma^null^, stock no. 005557) and Pmel transgenic mice (B6.Cg-Thy1^a^/Cy Tg(TcraTcrb)8Rest/J, stock no. 005023) were purchased from The Jackson Laboratory^71^. Mice at 7–12 weeks of age, both male and female, were used in all animal experiments, including T-cell isolation and tumour xenograft models. Littermate animals were randomized prior to experiments. All mice were kept in specific-pathogen-free conditions in the Animal Resource Center of the Research Institute at Nationwide Children’s Hospital and Baylor College of Medicine. Animal studies were approved by the Institutional Animal Care and Use Committee of the Research Institute at Nationwide Children’s Hospital (IACUC; protocol no. AR13–00055) and Baylor College of Medicine.

### In vivo imaging of tumour xenografts and T cells

For the LAN-1 xenograft, 1.5 × 10^6^ LAN-1 tumour cells were mixed in 100 μl 70% Matrigel (Corning) and were subcutaneously inoculated in the dorsal left and right flanks of 8-week-old female NSG mice. An aliquot of 8 × 10^6^ GD2-CAR T or GD2-CAR T–luciferase cells were i.v. injected into tumour-bearing mice when their tumour grew to about 4–6 mm in diameter (at around 6–8 d). For inosine treatments, inosine (Sigma-Aldrich) was administered (300 mg per kg (body weight)) by oral gavage daily after CAR-T-cell administration and throughout the experiment. Tumour volume (mm^3^) and overall survival were assessed daily throughout the experiment. For T-cell in vivo imaging, the images were captured using IVIS imaging system (Xenogen) after i.v. injection of 150 mg per kg (body weight) d-luciferin (Xenogen) at day 4 and day 7 after administration of GD2-CAR T–luciferase cells. Photon emission was analysed by constant region of interest, drawn over the tumour region, and the signal was measured as total photons per s per cm^2^ per steradian.

For the B16-F10 melanoma model, 8-week-old female C57BL/6 mice were inoculated with 1 × 10^5^ cells in the flank subcutaneously at day 0 and treated i.p. twice per week with anti-PDL1 antibody (200 μg). Inosine (300 mg per kg (body weight)) was administered by oral gavage daily, until animals reached the endpoint. Tumour volume (mm^3^) and overall survival were assessed daily throughout the experiment. To evaluate the tumour-infiltrating immune cells, after 15 d of inosine treatment, tumours, spleen and draining lymph nodes were dissected and dissociated using gentle MACS Dissociators, according to manufacturer’s instructions. For intracellular staining, cells were stimulated with PMA–ionomycin for 4 h, followed by cell surface staining; intracellular cytokine staining was performed in accordance with the manufacturer’s instructions using the Foxp3 permeabilization kit (eBioscience). After a 4-h incubation, cells were washed and incubated with surface antibodies for 30 min, and then cells were washed twice and incubated with fixation buffer for 2 h, followed by intracellular cytokines staining, which was performed for 45 min in permeabilization buffer. Flow cytometry analysis was performed on Novocyte (ACEA Biosciences) and results were analysed with FlowJo software (TreeStar). Additional details are provided in the Reporting Summary. In adoptive-transfer experiments, female C57BL/6 mice were inoculated with 1 × 10^5^ B16-F10 melanoma cells, and animals were irradiated sublethally (500 cGy) on day 6 after tumour-cell inoculation. On day 7, 4 × 10^6^ active Pmel^+^ T cells were i.v. injected, and inosine (300 mg per kg (body weight), oral gavage) was administered daily until animals reached the endpoint.

To establish the M3–9-M or TrampC2 xenograft model, 8-week-old male C57BL/6 mice were inoculated with 2 × 10^6^ M3–9-M cells or 1.5 × 10^6^ TrampC2 cells subcutaneously into the flanks. For the MC-38 xenograft, 8-week-old female C57BL/6 mice were inoculated subcutaneously in the flanks with 1 × 10^5^ MC-38 cells. In all these xenograft experiments, immunotherapy with control or PD-1/PDL1-blocking antibodies (200 μg) were performed i.p. twice per week, and inosine treatment was administered (300 mg per kg (body weight) in PBS) by oral gavage daily to the indicated experimental groups until the end of the experiment.

For all the animal studies, tumour sizes were measured daily by calliper at 6 d after implantation, and tumour volume was calculated by length × width^2^ × (π / 6) (ref. ^72^). For survival studies, animals were monitored daily until the endpoint was reached, that is when the experimental animals had (1) tumour diameter that reached 2 cm; (2) tumour ulceration reaching 1 cm in diameter, or persistent bleeding as a result of ulceration; (3) a 20% loss of body weight; (4) breathing difficulty or (5) poor mobility.

### Metabolite extraction and analysis by IC–UHR-FTMS and NMR

Cells were cultured in glucose-free medium with 2 mM [^13^C_6_]glucose (Cambridge Isotope Laboratories), or 2 mM [^13^C_5_]ribose-inosine (Omicron Biochemicals) in the absence or presence of inhibitor foro, or 2 mM [^13^C_5_]inosine and 2 mM [6,6-D_2_]glucose (Cambridge Isotope), respectively, for 24 h at 37 °C. Cells were then washed three times in cold PBS and underwent metabolic quenching in cold acetonitrile, and then were collected for metabolite extraction as described previously^73^. The polar extracts were reconstituted in Nanopure water before analysis on a Dionex ICS-5000+ ion chromatography system interfaced with a Thermo Fusion Orbitrap Tribrid mass spectrometer (Thermo Fisher Scientific), as previously described^74^, using a *m/z* scan range of 80–700. Peak areas were integrated and exported to Microsoft Excel via the Thermo TraceFinder (version 3.3) software package before natural-abundance correction^75^. The isotopologue distributions of metabolites were calculated as the mole fractions, as previously described^76^. The number of moles of each metabolite was determined by calibration of the natural-abundance-corrected signal against that of authentic external standards. The amount was normalized to the amount of extracted protein, and is reported in nmol per mg protein.

Polar extracts reconstituted in D_2_O (>99.9%, Cambridge Isotope Laboratories) containing 0.5 mmol l^–1^ d_6_-2,2-dimethyl-2-silapentane-5-sulfonate (DSS) as internal standard were analysed by 1D ^1^H and (^1^H,^13^C)-HSQC NMR on a 14.1 T DD2 NMR spectrometer (Agilent Technologies). 1D ^1^H spectra were acquired using the standard PRESAT pulse sequence with 512 transients, 16,384 data points, 12 parts per million (ppm) spectral width, an acquisition time of 2 s and a 6-s recycle time with weak irradiation on the residual HOD signal during the relaxation delay. The raw fids were zero-filled to 131,072 points and apodized with 1-Hz exponential line broadening prior to Fourier transformation. 1D HSQC spectra were recorded with an acquisition time of 0.25 s with GARP decoupling, and a recycle time of 2 s over a spectral width of 12 ppm, with 1024 transients. The HSQC spectra were then apodized with unshifted Gaussian function and 4-Hz exponential line broadening, and were zero-filled to 16,384 data points before Fourier transformation. Metabolites were assigned by comparison with in-house^77^ and public NMR databases. Metabolites and their ^13^C isotopomers were quantified using the MesReNova software (Mestrelab) by peak deconvolution. The peak intensities of metabolites obtained were converted into molar mass by calibration against the peak intensity of DSS (27.5 nmol) at 0 ppm for ^1^H spectra and that of phosphocholine at 3.21 ppm (molar mass determined from 1D ^1^H spectra) for HSQC spectra before normalization with mg protein in each sample.

### siRNA transfection in HeLa cells

The siRNA oligonucleotides corresponding to human PNP (purine nucleoside phosphorylase) were purchased from Fisher. siRNA oligonucleotides (20 nM) were transfected into HeLa cells using Lipofectamine RNAiMAX reagent (Invitrogen). After 72 h of transfection, immunoblots were carried out to examine the targeted protein knockdown.

### Electroporation of human T cells

PBMCs were stimulated with plate-bound anti-CD3 and anti-CD28 antibodies for 2 d before electroporation. Cells were centrifuged and washed once with PBS. An aliquot of 1 × 10^6^ cells was resuspended in 100 μl electroporation buffer T (Thermo Fisher Scientific), and 100 nM siRNA oligonucleotides corresponding to human PNP was added. Electroporation was performed at 2,150 V, 20-ms and 1 pulse settings for stimulated PBMCs using Neon electroporation device (Thermo Fisher Scientific). Immediately after electroporation, the cells were plated in a 12-well plate with 100 U ml^–1^ IL-2 and were incubated at 37 °C.

### qPCR and western blot

Total RNA was isolated using the Quick-RNA MiniPrep Kit (Zymo Research) and was reverse transcribed using random hexamer and M-MLV Reverse Transcriptase (Invitrogen). SYBR green-based quantitative RT–PCR was performed using the BIO-RAD CFX96 Real-Time System. The relative gene expression was determined by the comparative *C*_T_ method, also referred to as the $2^{-\Delta\Delta{C_T}}$ method. The data are presented as the fold change in gene expression normalized to an internal reference gene (β2-microglobulin) and relative to the control (the first sample in the group). Samples for each experimental condition were run in triplicate PCR reactions. Primer sequences were obtained from PrimerBank^78^. The following pair of primers were used for detecting mouse PNP (5′-ATCTGTGGTTCCGGCTTAGGA-3′ and 5′-TGGGGAAAGTTGGGTATCTCAT-3′).

Cells were collected, lysed and sonicated at 4 °C in a lysis buffer (50 mM Tris-HCl, pH 7.4, 150 mM NaCl, 0.5% SDS, 5 mM sodium pyrophosphate, protease and phosphatase inhibitor tablet). Cell lysates were centrifuged at 13,000*g* for 15 min, and the supernatant was recovered. The protein concentrations were determined by using the Pierce BCA Protein Assay Kit (Thermo Fisher Scientific). After 5-min boiling in 4× NuPAGE LDS Sample Buffer with 10× reducing solution (Thermo Fisher Scientific), the proteins were separated by NuPAGE 4–12% Protein Gels (Thermo Fisher Scientific), transferred to PVDF membranes by using the iBlot Gel Transfer Device (Thermo Fisher Scientific) and probed with the appropriate primary antibodies. Membrane-bound primary antibodies were detected using secondary antibodies conjugated with horseradish peroxidase. Immunoblots were developed on films using the enhanced chemiluminescence technique.

### The extracellular acidification rate and OCR

The extracellular acidification rate (ECAR) and OCR for mitochondrial oxidation were evaluated using the pH-Xtra assay (Agilent Technologies) and mitoXpress assay (Agilent Technologies), respectively. The manufacturer’s recommended procedures were followed. Human PBMCs, activated by plate-bound anti-CD3 and anti-CD28 antibodies for at least 3 d, were plated at 600,000 cells per well using fresh medium containing 4 mM glucose or 4 mM inosine. For ECAR, cells were incubated in the absence of CO_2_ for 2 h prior to the assay. Respiration buffer was customized by supplementing glucose or inosine. Both assays were read during 180 min at 37 °C in a Cytation 1 Cell Imaging Multi-Mode Reader (Biotek Instruments).

### Metabolic assay

The fatty acid β-oxidation rate was determined by measuring the detritiation of [9,10-^3^H]palmitic acid^79,80^. One million T cells were suspended in 0.5 ml fresh medium. The experiment was initiated by adding 3 μCi [9,10-^3^H]palmitic acid in complex with 5% BSA (lipids free; Sigma-Aldrich) and, 2 h later, media were transferred to 1.5-ml microcentrifuge tubes containing 50 μl 5N HCl. The microcentrifuge tubes were then placed in 20-ml scintillation vials containing 0.5 ml water, and vials were capped and sealed. ^3^H_2_O was separated from unmetabolized [9,10-^3^H]palmitic acid by evaporation diffusion for 24 h at room temperature. A cell-free sample containing 3 μCi [9,10-^3^H]palmitic acid was included as a background control.

Glutamine oxidation activity was determined by the rate of ^14^CO_2_ released from [U-^14^C]glutamine^81^. In brief, one million T cells were suspended in 0.5 ml fresh medium. To facilitate the collection of ^14^CO_2_, cells were dispensed into 7-ml glass vials (TS-13028, Thermo) with a PCR tube containing 50 μl 0.2 M KOH glued on the sidewall. After addition of 0.5 μCi [U-^14^C]glutamine, the vials were capped using a screw cap with rubber septum (TS-12713, Thermo). The assay was stopped 2 h later by injection of 100 μl 5 N HCl, and the vials were kept at room temperate overnight to trap the ^14^CO_2_. The 50 μl KOH in the PCR tube was then transferred to scintillation vials containing 10 ml scintillation solution for counting. A cell-free sample containing 0.5 μCi [U-^14^C]glutamine was included as a background control.

### Nucleoside-uptake assay

PBMCs were stimulated with plate-bound anti-CD3 and anti-CD28 antibodies for 3 d. Then, 1 × 10^7^ active human T cells were suspended in 600 μl medium containing 2 mM cold inosine or adenosine and 20 nM of the same radioactive-labelled nutrient ([2,8-^3^H]inosine or [2,8-^3^H]adenosine), and were incubated at 37 °C for 10 min. Aliquots of 200 μl cell suspension were loaded onto separation-buffer layers, which were prepared by adding 100 μl 20% perchloric acid/8% sucrose solution to the bottom of a 1.5-ml microcentrifuge tube, and were overlaid with 800 μl 1-bromododecane. The tubes containing cell suspension and separation-buffer layers were centrifuged at 15,000*g* for 1 min. The tube was then snap-frozen in a dry-ice bath, and the tip of the tube (bottom layer with cell lysis) just above the perchloric acid–sucrose–bromododecane interface was cut off and transferred into 1 well of a 24-well plate. The tip was then washed with 300 μl 0.5% SDS/1% Triton X-100. The solution collected from each tube was then transferred to a scintillation vial containing 10 ml scintillation solution for counting.

### Statistical analysis

Statistical analysis was conducted using the GraphPad Prism software (GraphPad Software). Two-way ANOVA was used to analyse differences between tumour progression curves across all the animal experiments. Kaplan–Meier curves and corresponding log-rank Mantel–Cox tests were used to evaluate the statistical differences between groups in survival studies. Unpaired two-tailed Student’s *t*-test was used to assess differences in other experiments. *P* values smaller than 0.05 were considered significant, with *P* values < 0.05, *P* values < 0.01 and *P* values < 0.001 indicated as *, ** and ***, respectively.

### Reporting Summary

Further information on research design is available in the Nature Research Reporting Summary linked to this article.

## Supplementary information

Supplementary Information Supplementary Figs. 1–3 and Supplementary Tables 1 and 2

Reporting Summary

Supplementary Data 1 Supplementary Dataset for Supplementary Fig. 1b,c.

Supplementary Data 2 Supplementary Dataset for Supplementary Fig. 2a,b.

## Data Availability

The data that support the findings of this study are available from the corresponding author (R.W.) upon request. Source data for Figs. 1–6, Extended Data Figs.1–10 and Supplementary Figs. 1 and 2 are provided with the paper.

## Source data

Source Data Fig. 1 Statistical Source Data
Source Data Fig. 2 Statistical Source Data
Source Data Fig. 3 Statistical Source Data
Source Data Fig. 3 Unprocessed Western Blots
Source Data Fig. 4 Statistical Source Data
Source Data Fig. 5 Statistical Source Data
Source Data Fig. 6 Statistical Source Data
Source Data Extended Data Fig. 1 Statistical Source Data
Source Data Extended Data Fig. 2 Statistical Source Data
Source Data Extended Data Fig. 3 Statistical Source Data
Source Data Extended Data Fig. 4 Statistical Source Data
Source Data Extended Data Fig. 5 Statistical Source Data
Source Data Extended Data Fig. 6 Statistical Source Data
Source Data Extended Data Fig. 6 Unprocessed Western Blots
Source Data Extended Data Fig. 7 Statistical Source Data
Source Data Extended Data Fig. 8 Statistical Source Data
Source Data Extended Data Fig. 8 Unprocessed Western Blots
Source Data Extended Data Fig. 9 Statistical Source Data
Source Data Extended Data Fig. 10 Statistical Source Data
