# Supplementary material for: Inosine is an alternative carbon source for CD8+-T-cell function under glucose restriction
Source: Nat Metab. 2020 Jun 15;2(7):635–47. doi: 10.1038/s42255-020-0219-4 (PMC7371628; doi:10.1038/s42255-020-0219-4)
Supplement: Supplementary file 1 — Supplementary Figs. 1–3 and Supplementary Tables 1 and 2 [file 42255_2020_219_MOESM1_ESM.pdf]

nature metabolism

SUPPLEMENTARY INFORMATION

https://doi.org/10.1038/s42255-020-0219-4

In the format provided by the authors and unedited.

Inosine is an alternative carbon source for CD8^{+}-T-cell function under glucose restriction

Tingting Wang[1,8], J. N. Rashida Gnanaprakasam[1,8], Xuyong Chen[1], Siwen Kang[1], Xuequn Xu[1], Hua Sun[2], Lingling Liu[1], Hayley Rodgers[1], Ethan Miller[1], Teresa A. Cassel[3], Qiushi Sun[3], Sara Vicente-Muñoz[3], Marc O. Warmoes[3], Penghui Lin[3], Zayda Lizbeth Piedra-Quintero[4], Mireia Guerau-de-Arellano[4], Kevin A. Cassady[1], Song Guo Zheng[5], Jun Yang[6], Andrew N. Lane[3], Xiaotong Song[2,7] ✉, Teresa W.-M. Fan[3] ✉ and Ruoning Wang[1] ✉

[1]Center for Childhood Cancer & Blood Diseases, Hematology/Oncology & BMT, Abigail Wexner Research Institute at Nationwide Children's Hospital, Ohio State University, Columbus, OH, USA. [2]The Center for Cell and Gene Therapy, Baylor College of Medicine, Houston, TX, USA. [3]Center for Environmental and Systems Biochemistry, Department of Toxicology and Cancer Biology, Markey Cancer Center, University of Kentucky, Lexington, KY, USA. [4]School of Health and Rehabilitation Sciences, Division of Medical Laboratory Science, College of Medicine, Wexner Medical Center, Ohio State University, Columbus, OH, USA. [5]Division of Rheumatology and Immunology, Department of Internal Medicine at Ohio State University of Medicine and Wexner Medical Center, Columbus, OH, USA. [6]Department of Surgery, St Jude Children's Research Hospital, Memphis, TN, USA. [7]Icell Kealex Therapeutics, Houston, TX, USA. [8]These authors contributed equally: T. Wang, J. N. R. Gnanaprakasam. ✉e-mail: shautong.song@icellkealex.com; twmfan@gmail.com; ruoning.wang@nationwidechildrens.org

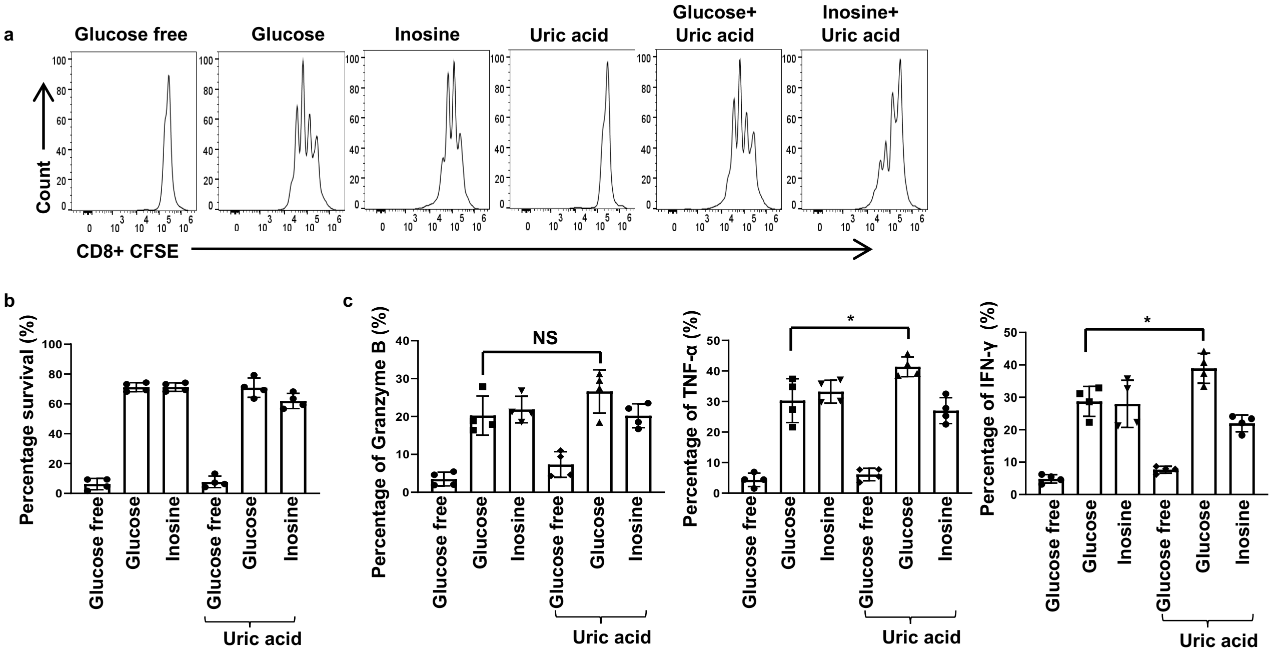

Supplementary Figure 1. Uric acid is not the downstream effector metabolite of inosine. (a-c) Naive CD8+ T cells from C57BL/6 mice were activated by plate bound anti-CD3 and anti-CD28 antibodies and differentiated in the indicated conditional media in the presence and absence of uric acid (1mM) for 4 days. (a) Cell proliferation (CFSE), (b) cell survival (7AAD), and (c) indicated intracellular effector molecules were determined by FACS. Data are presented as mean ± SD (n=4). (c) NS, P=0.1477; *, P=0.0307 and 0.0205 for Granzyme B, TNF-α, and IFN-γ for Glucose versus Glucose+Uric acid by unpaired two-tailed t-test. Data are representative of three independent experiments. Sample size (n) represents biologically independent samples (b-c).

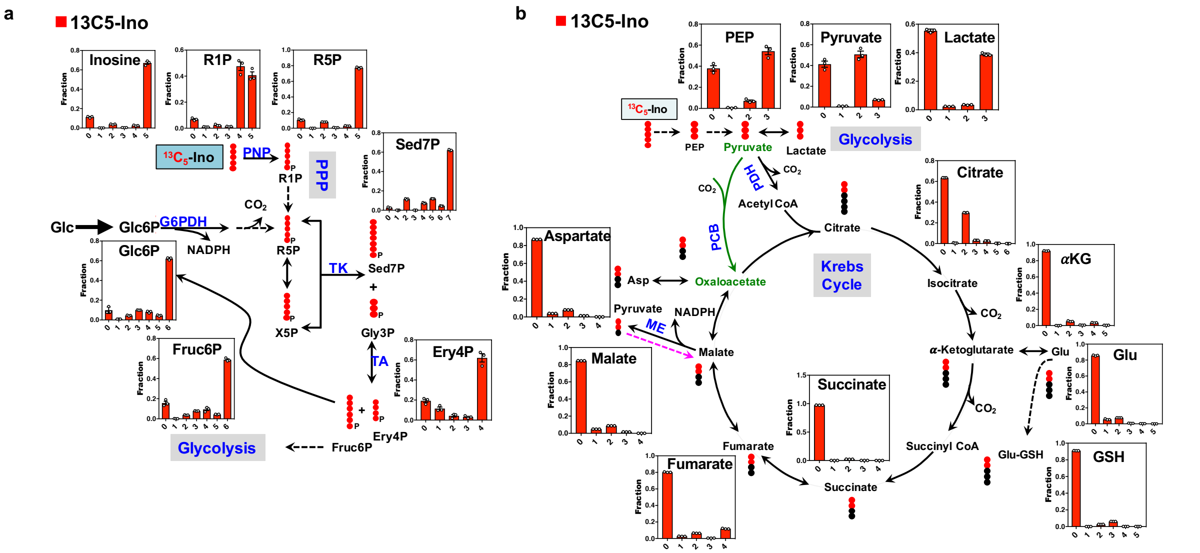

Supplementary Figure 2. Incorporation of the ribose subunit of inosine into the central carbon metabolism in the presence of glucose in human T effector cells. Active human T cells were incubated in media containing both [$^{13}C_5$]-Inosine and [6,6-D_2]-Glucose for 24 h, extracted as described in methods, and analyzed for the incorporation of [$^{13}C_5$]-Ino in the presence of glucose into PPP metabolites (a) and glycolytic/Krebs cycle metabolites (b) by IC-UHR-FTMS. Data are presented as mean ± SEM (n=3). All symbols and abbreviations are as described in **Figure 2**. Numbers in the X-axis represent those of ^{13}C atoms in given metabolites.

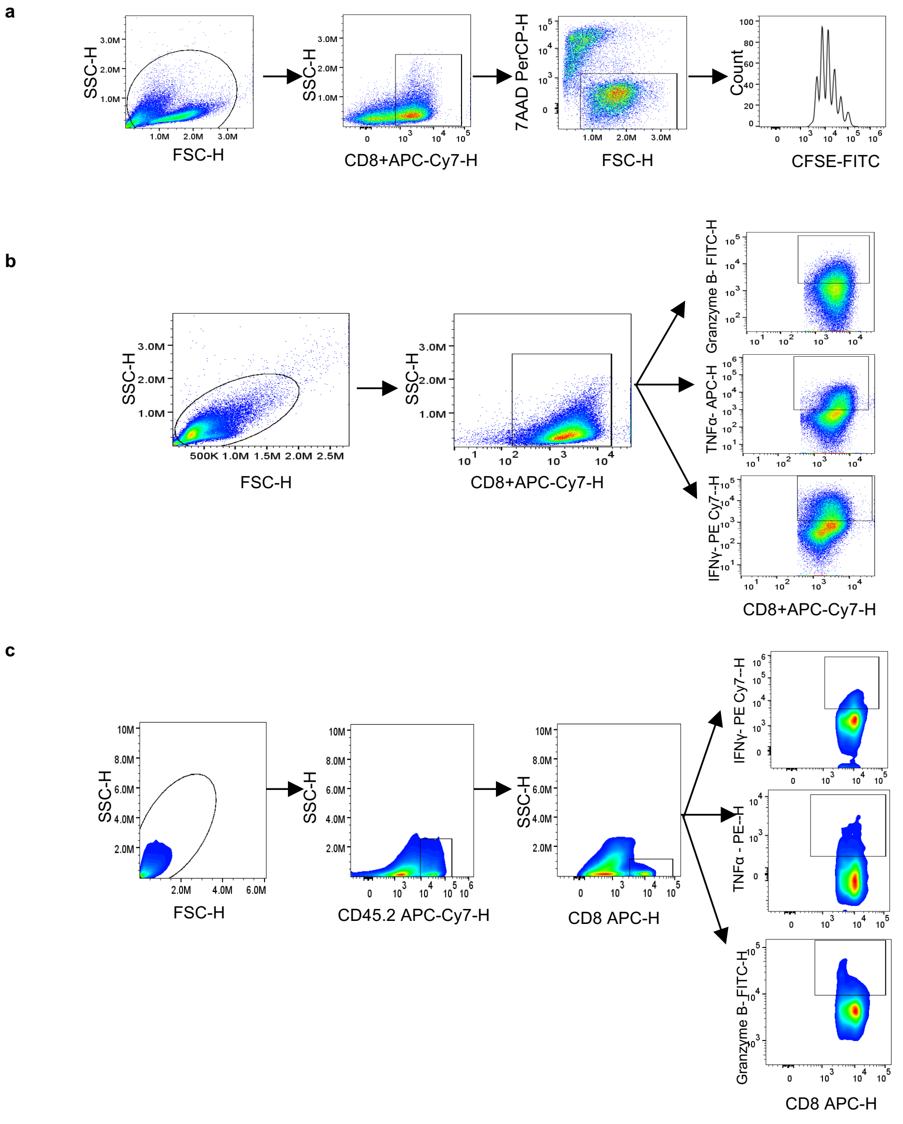

Supplementary Figure 3. Gating strategy for analysis of (a) CD8+ T cell proliferation (b) intracellular cytokines and effector molecule expression (c) tumor infiltrated effector T cells and intracellular cytokines.

Supplementary Table 1

**Antibody**	**Cat#/Vendor**
Mouse anti-CD3 mAb	BE0001-1, BioXcell
Mouse anti-CD28 mAb	BE0015-1, BioXcell
Anti mouse CD8-APC-Cy7	100714, Biolegend
Anti human/mouse Granzyme B-FITC	515403, Biolegend
Anti mouse TNF-α- APC	17-7321-82, eBioscience
Anti mouse IFN-γ- PE-Cy7	25-7319-82, eBioscience
Anti-human CD8a Antibody - APC-Cy7	300926, Biolegend
Anti-human TNF-α -PE	502909, Biolegend
Anti-human IFN-γ- PE-Cy7	506518, Biolegend
Anti mouse CD45.2 - PerCP-Cyanine5.5	45-0454-82, eBioscience
InVivoMAb anti m PD-L1	BE0101, BioXcell
InVivoMAb anti m PD-1	BE0146, BioXcell
Anti-human CD3 (OKT-3)	BE0001, BioXcell
Anti-human/monkey CD28.2	BE0291, BioXcell
anti-PNP	sc-271890, Santa Cruz
anti-actin	sc-47778, Santa Cruz
**Reagents**	**Cat#/Vendor**
Recombinant human IL-2	200-02, Peprotech
Recombinant murine IL-2	212-12, Peprotech
Cell Stimulation Cocktail (plus protein transport inhibitors) (500X)	00-4975-93,eBioscience
carboxyfluorescein diacetate succinimidyl ester(CFSE)	Invitrogen
7-amino-actinomycin D(7AAD)	420404,Biolegend
D-(+)-Glucose	G7021, Sigma-Aldrich
Inosine	I4125, Sigma-Aldrich
Adenosine	164040050, ACROS
D-(+)-Glucose	G7021, Sigma-Aldrich
RPMI 1640 Medium, No Glucose	11-879-020, Gibco
Retronectin	T100, Takara/clontech
Human gp100	RP20344, GenScript
Forodesine Hydrochloride	HY-16209, MedChem Express
$^{13}C_6$-Glucose	CLM-1396, Cambridge Isotope Lab
$^{13}C_5$-Inosine	NUC-072, OMICRON
D-GLUCOSE (6,6-D2, 99%)	DLM-349, Cambridge Isotope Lab
[$^{14}C_5$]-glutamine	ART0115, American Radiolabeled Chemicals
[9,10-^{3}H]-palmitic acid	MT 845, Moravek
[2,8-^{3}H]-inosine	MT 678, Moravek
[2,8-^{3}H]-adenosine	MT 793, Moravek
Etomoxir	11969, Caymen
Methotrexate	13960100, Caymen
Calcein AM	C3099, Invitrogen
Sodium pyruvate solution	S8636, Sigma-Aldrich
Uric Acid	69-93-2, Alfa Aesar
hypoxathine	H9636, Sigma-Aldrich
ribose-1-phosphate	83866, Sigma-Aldrich
perchloric acid	311413, Sigma-Aldrich
sucrose	S7930, Sigma-Aldrich
1-Bromododecane	106912500, ACROS
CD45R(B220) microbeads, mouse	5150309030, Miltenyi Biotec
Naive CD8+ T cell isolation kit,mouse	130-096-543, Miltenyi Biotec
MojoSort™ Mouse CD8 Naive T Cell Isolation Kit	480044, Biolegend
MojoSort™ Human CD3 T Cell Isolation Kit	480021, Biolegend

Supplementary Table 2

Data sets	Asterisks	P value (13C5-Ino versus 13C5-Ino+Foro)
13C2-Citrate	***	8.18755E-05
13C3-Citrate	***	2.96234E-06
13C4-Citrate	***	2.15878E-06
13C5-Citrate	***	4.70221E-06
13C6-Citrate	***	6.49864E-06
13C2-αKG	**	0.00431901
13C3-αKG	**	0.001316994
13C4-αKG	***	0.000277638
13C5-αKG	***	0.000550666
13C1-Glu	***	6.64732E-06
13C2-Glu	***	7.0498E-11
13C3-Glu	***	8.341E-12
13C4-Glu	***	3.41648E-10
13C5-Glu	***	1.6913E-09
13C1-GSH	***	1.63113E-05
13C2-GSH	***	1.71179E-10
13C3-GSH	***	4.47E-13
13C4-GSH	***	4.553E-12
13C5-GSH	***	2.52106E-10
13C1-Succinate	*	0.018325258
13C2-Succinate	***	0.000546566
13C3-Succinate	***	0.00069771
13C4-Succinate	**	0.00218597
13C1-Fumarate	***	4.94266E-05
13C2-Fumarate	***	9.47052E-08
13C3-Fumarate	***	8.385E-12
13C4-Fumarate	***	3.1535E-09
13C1-Malate	***	2.37424E-05
13C2-Malate	***	1.97296E-07
13C3-Malate	***	6.0447E-08
13C4-Malate	***	2.33909E-08
13C1-Aspartate	***	6.72424E-08
13C2-Aspartate	***	7.3491E-11
13C3-Aspartate	***	9.183E-12
13C4-Aspartate	***	1.32059E-10
