# Supplementary material for: Inosine is an alternative carbon source for CD8+-T-cell function under glucose restriction
Source: Nat Metab. 2020 Jun 15;2(7):635–47. doi: 10.1038/s42255-020-0219-4 (PMC7371628; doi:10.1038/s42255-020-0219-4)
Supplement: Supplementary file 2 — Reporting Summary [file 42255_2020_219_MOESM2_ESM.pdf]

nature research

Corresponding author(s): Ruoning Wang
Last updated by author(s): 4/9/2020

Reporting Summary

Nature Research wishes to improve the reproducibility of the work that we publish. This form provides structure for consistency and transparency in reporting. For further information on Nature Research policies, see Authors & Referees and the Editorial Policy Checklist.

Statistics

For all statistical analyses, confirm that the following items are present in the figure legend, table legend, main text, or Methods section.

n/a	Confirmed	
☐	☒	The exact sample size (*n*) for each experimental group/condition, given as a discrete number and unit of measurement
☐	☒	A statement on whether measurements were taken from distinct samples or whether the same sample was measured repeatedly
☐	☒	The statistical test(s) used AND whether they are one- or two-sided<br>*Only common tests should be described solely by name; describe more complex techniques in the Methods section.*
☒	☐	A description of all covariates tested
☒	☐	A description of any assumptions or corrections, such as tests of normality and adjustment for multiple comparisons
☐	☒	A full description of the statistical parameters including central tendency (e.g. means) or other basic estimates (e.g. regression coefficient) AND variation (e.g. standard deviation) or associated estimates of uncertainty (e.g. confidence intervals)
☐	☒	For null hypothesis testing, the test statistic (e.g. *F*, *t*, *r*) with confidence intervals, effect sizes, degrees of freedom and *P* value noted<br>*Give P values as exact values whenever suitable.*
☒	☐	For Bayesian analysis, information on the choice of priors and Markov chain Monte Carlo settings
☒	☐	For hierarchical and complex designs, identification of the appropriate level for tests and full reporting of outcomes
☒	☐	Estimates of effect sizes (e.g. Cohen's *d*, Pearson's *r*), indicating how they were calculated

Our web collection on statistics for biologists contains articles on many of the points above.

Software and code

Policy information about availability of computer code

Data collection	NovoExpress® Software, IncuCyte ZOOM
Data analysis	Flowjo V10, Graphpad Prism 8, IncuCyte ZOOM

For manuscripts utilizing custom algorithms or software that are central to the research but not yet described in published literature, software must be made available to editors/reviewers. We strongly encourage code deposition in a community repository (e.g. GitHub). See the Nature Research guidelines for submitting code & software for further information.

Data

Policy information about availability of data

All manuscripts must include a data availability statement. This statement should provide the following information, where applicable:

- Accession codes, unique identifiers, or web links for publicly available datasets
- A list of figures that have associated raw data
- A description of any restrictions on data availability

Data and materials supporting the findings of this study are available within the article and its supplementary information files.

Field-specific reporting

Please select the one below that is the best fit for your research. If you are not sure, read the appropriate sections before making your selection.

☒ Life sciences ☐ Behavioural & social sciences ☐ Ecological, evolutionary & environmental sciences

For a reference copy of the document with all sections, see nature.com/documents/nr-reporting-summary-flat.pdf

Life sciences study design

All studies must disclose on these points even when the disclosure is negative.

Sample size	No sample size calculations were performed since sample size were chosen based on the previous experience and publications.
Data exclusions	Figure 6c, five tumor data was excluded for the in vivo CAR T experiment, due to the leakage of Lan-1 cells during inoculation or unsuccessful tail vein injection of CAR T cells to NSG mice. Extended Data Figure 10, five, four, and five tumor data were excluded due to the leakage of tumor cell inoculation for MC-38, TrampC2, and M3-9-M mouse models, respectively.
Replication	All experiments were conducted with at least two independent experiments and multiple biological replicates.
Randomization	We used age and gender matched animals for all the experiments. Litter-mate animals were randomized prior to experiments.
Blinding	Experiments were not performed blinded because all analysis were performed using same gating as control under the same condition.

Reporting for specific materials, systems and methods

We require information from authors about some types of materials, experimental systems and methods used in many studies. Here, indicate whether each material, system or method listed is relevant to your study. If you are not sure if a list item applies to your research, read the appropriate section before selecting a response.

Materials & experimental systems

n/a	Involved in the study	
☐	☒	Antibodies
☐	☒	Eukaryotic cell lines
☒	☐	Palaeontology
☐	☒	Animals and other organisms
☒	☐	Human research participants
☒	☐	Clinical data

Methods

n/a	Involved in the study	
☒	☐	ChIP-seq
☐	☒	Flow cytometry
☒	☐	MRI-based neuroimaging

Antibodies

Antibodies used	Mouse anti-CD3 mAb (BioXcell), Mouse anti-CD28 mAb (BioXcell), Anti mouse CD8-APC-Cy7 (Biolegend), Anti human/mouse Granzyme B-FITC (Biolegend), Anti mouse TNF-α- APC (eBioscience), Anti mouse IFN-γ- PE-Cy7 (eBioscience), Anti-human CD8a Antibody - APC-Cy7 (Biolegend), Anti-human TNF-α -PE (Biolegend), Anti-human IFN-γ- PE-Cy7 (Biolegend), Anti mouse CD45.2 - PerCP-Cyanine5.5 (eBioscience), InVivoMAb anti m PD-L1 (BioXcell), InVivoMAb anti m PD-1 (BioXcell), Anti-human CD3 (OKT-3) (BioXcell), Anti-human/monkey CD28.2 (BioXcell), anti-PNP (Santa Cruz), anti-Actin (Santa Cruz).
Validation	All antibodies are commercially available and validated for indicated applications and passed QC controls.

Eukaryotic cell lines

Policy information about cell lines

Cell line source(s)	ATCC, other research groups
Authentication	Cell lines used in this study were purchased from ATCC or from other research groups and were not authenticated.
Mycoplasma contamination	Cell lines used in this study were not tested for Mycoplasma contamination.
Commonly misidentified lines (See ICLAC register)	*Name any commonly misidentified cell lines used in the study and provide a rationale for their use.*

Animals and other organisms

Policy information about studies involving animals; ARRIVE guidelines recommended for reporting animal research

Laboratory animals	C57BL/6NHsd , NSG (NOD-scid IL2Rgammanull), and Pmel transgenic mice (B6.Cg-Thy1a/Cy Tg(TcraTcrb)8Rest). Mice were 7-12 weeks old when performing the experiments.
Wild animals	This study does not include any wild animal.
Field-collected samples	This study does not include samples collected from the field.

Ethics oversight	Animal protocols were approved by the Institutional Animal Care and Use Committee of the Research Institute at Nationwide Children's Hospital and Baylor College of Medicine.

Note that full information on the approval of the study protocol must also be provided in the manuscript.

Flow Cytometry

Plots

Confirm that:

- [ ] The axis labels state the marker and fluorochrome used (e.g. CD4-FITC).
- [x] The axis scales are clearly visible. Include numbers along axes only for bottom left plot of group (a 'group' is an analysis of identical markers).
- [x] All plots are contour plots with outliers or pseudocolor plots.
- [x] A numerical value for number of cells or percentage (with statistics) is provided.

Methodology

Sample preparation	Spleen and lymph nodes were passed through 70 micron filters. For analysis of surface markers, cells were stained in PBS containing 2% (w/v) BSA and the appropriate antibodies.
Instrument	Novocyte
Software	Flowjo V10
Cell population abundance	Not applicable
Gating strategy	Gating strategy is shown in Supplementary Figure 3 for analysis of (a) CD8+ T cell proliferation (b) intracellular cytokines and effector molecule expression (c) tumor infiltrated effector T cells and intracellular cytokines, respectively. FSC-SSC-H gating was used as preliminary gating for lymphocyte population followed by analysis of effector T cells (CD8+) in all the experiments. For analysis of T cell infiltration in tumors, gating strategy for flow cytometry analysis was performed by gating for leucocytes (CD45 +), and then effector T cells (CD8+), followed by analysis of intracellular effector molecules.

- [x] Tick this box to confirm that a figure exemplifying the gating strategy is provided in the Supplementary Information.
