# Supplementary figures and images for: Inosine is an alternative carbon source for CD8+-T-cell function under glucose restriction
Source: Nat Metab. 2020 Jun 15;2(7):635–47. doi: 10.1038/s42255-020-0219-4 (PMC7371628; doi:10.1038/s42255-020-0219-4)

PNP

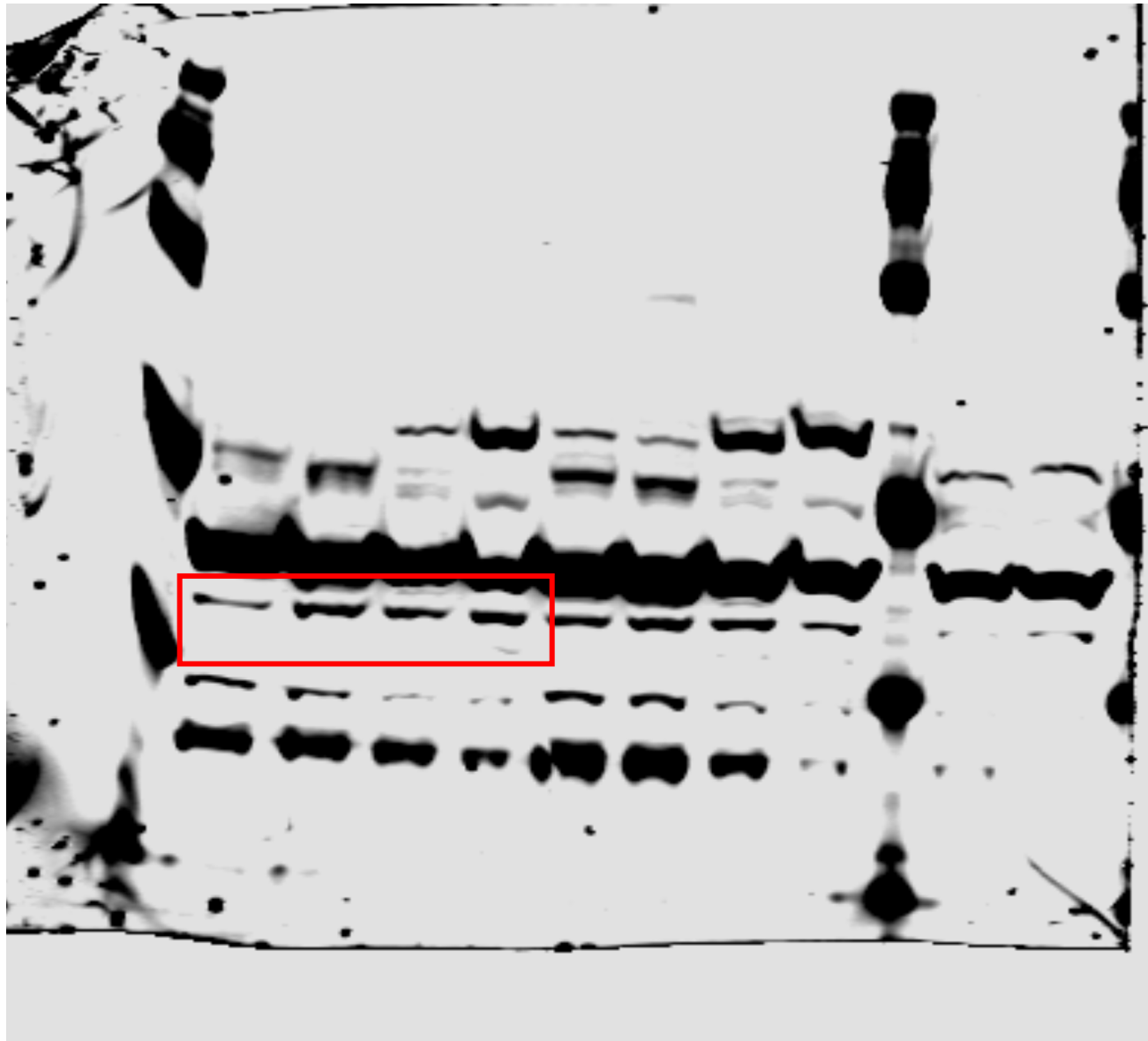

## Actin

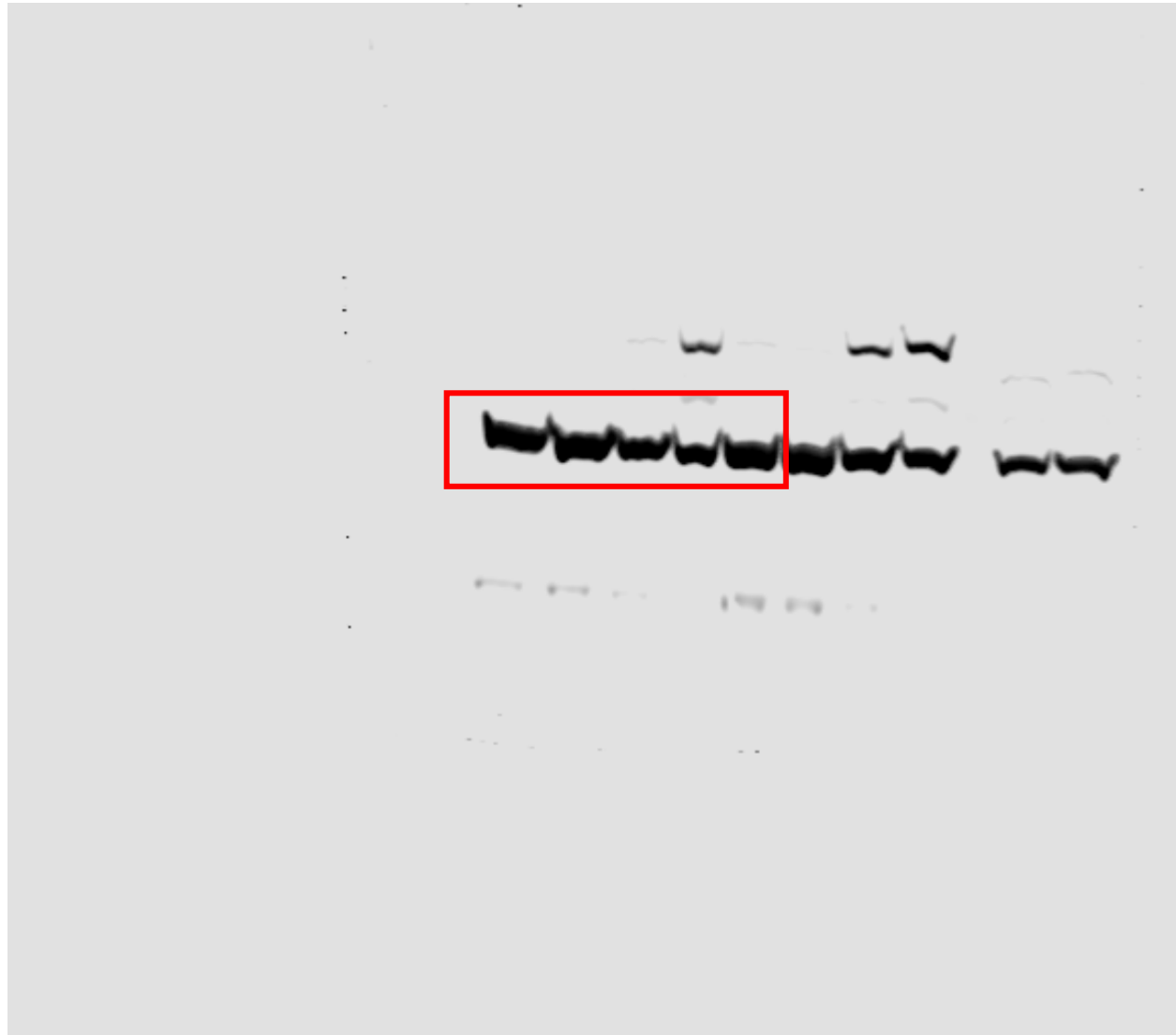

Supplement: Source Data Fig. 3 — Unprocessed Western Blots [file 42255_2020_219_MOESM8_ESM.pdf]

81-51-5

...FUJI-HRC-(SAFETY)...

...FUJI-HRC-(SAFETY)...

...

2/14/mb gel 1

PNP

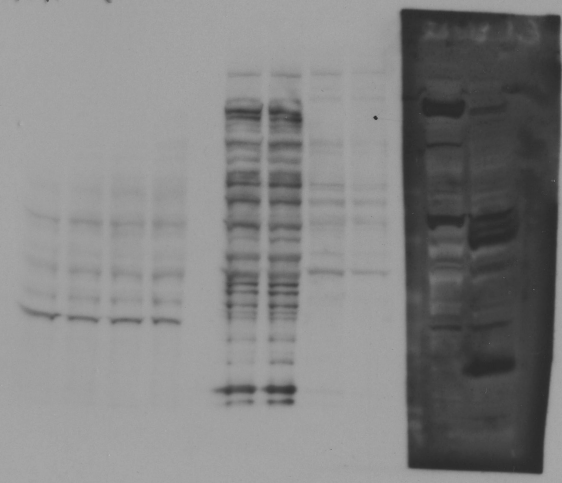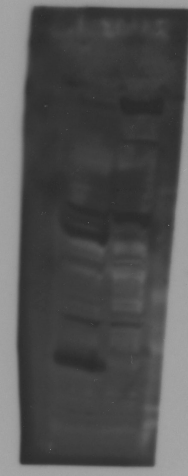

← gus

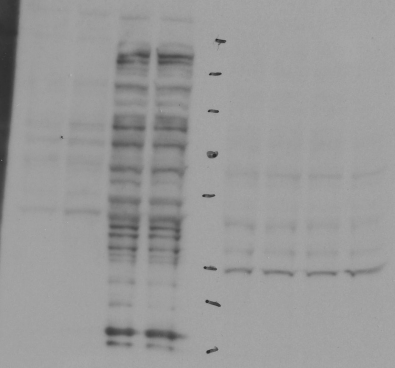

gel 2

PNP

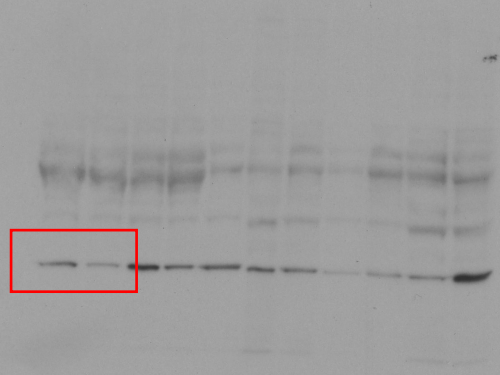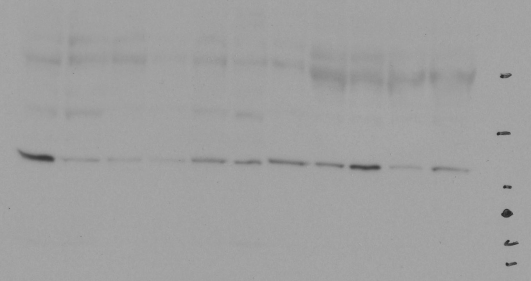

7.19.18  $\beta$ -actin

7/19/18  
862

Actin

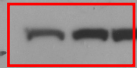

7/16/18 gel H

12

13

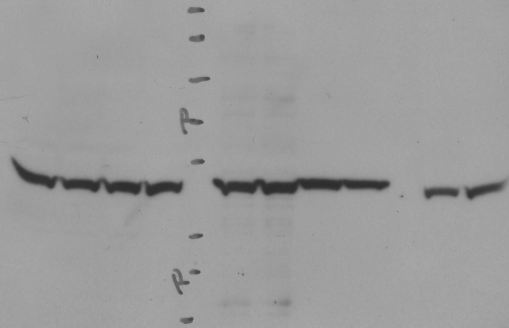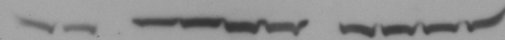

Supplement: Source Data Extended Data Fig. 6 — Unprocessed Western Blots [file 42255_2020_219_MOESM18_ESM.pdf]
