# Supplementary material for: Inosine is an alternative carbon source for CD8+-T-cell function under glucose restriction
Source: Nat Metab. 2020 Jun 15;2(7):635–47. doi: 10.1038/s42255-020-0219-4 (PMC7371628; doi:10.1038/s42255-020-0219-4)
Supplement: Source Data Extended Data Fig. 8 — Unprocessed Western Blots [file 42255_2020_219_MOESM21_ESM.pdf]

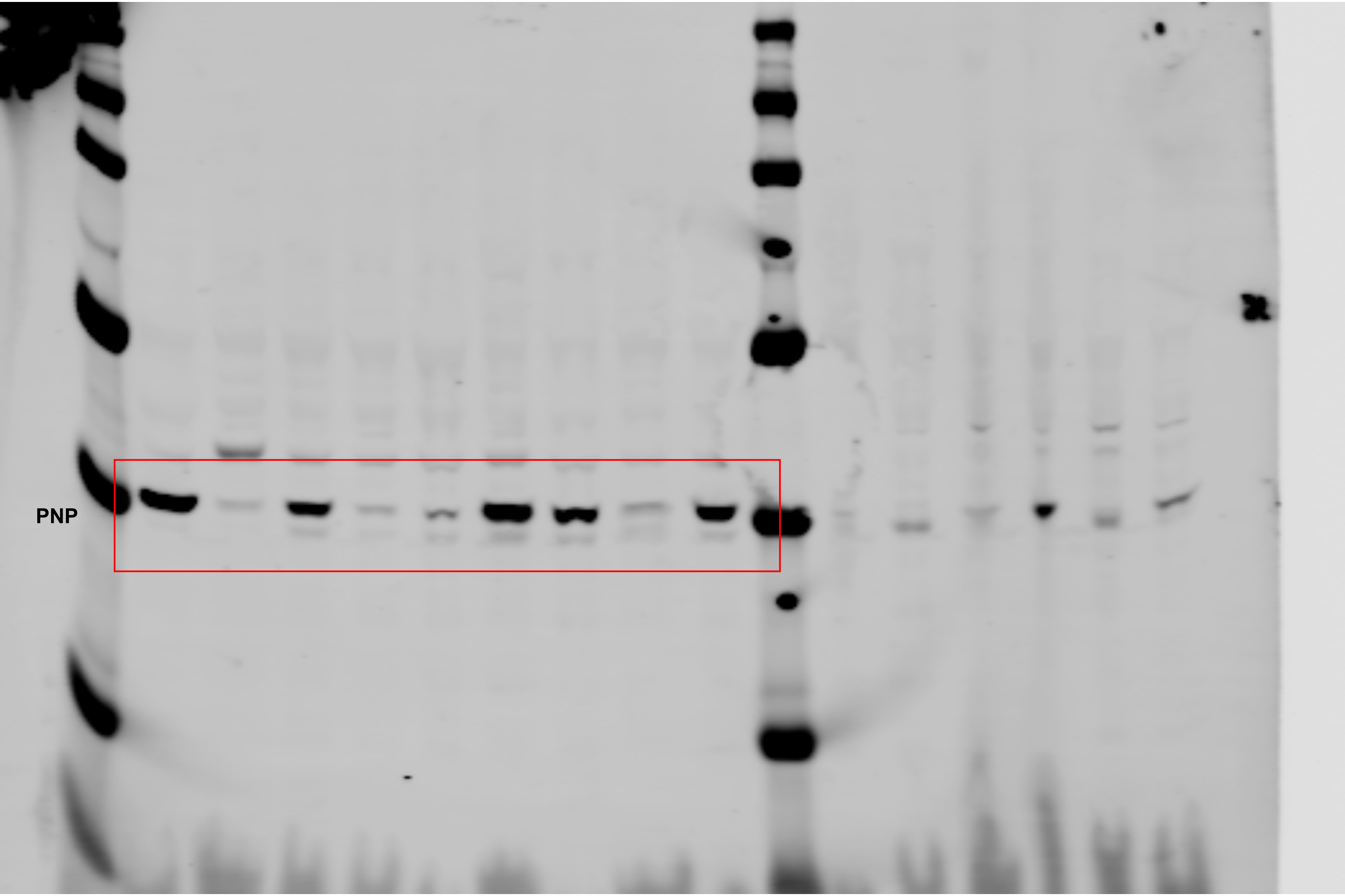
PNP

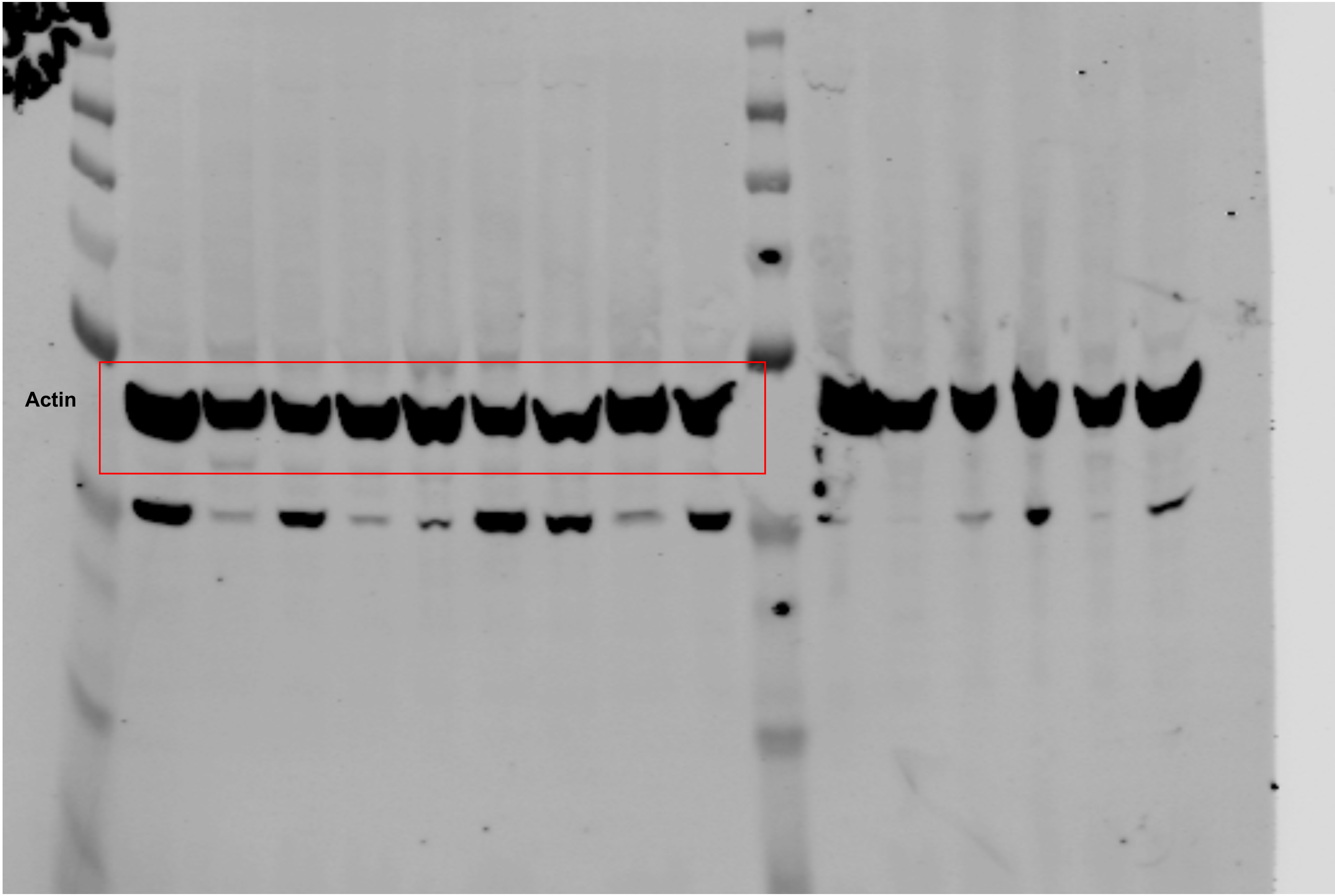
Actin

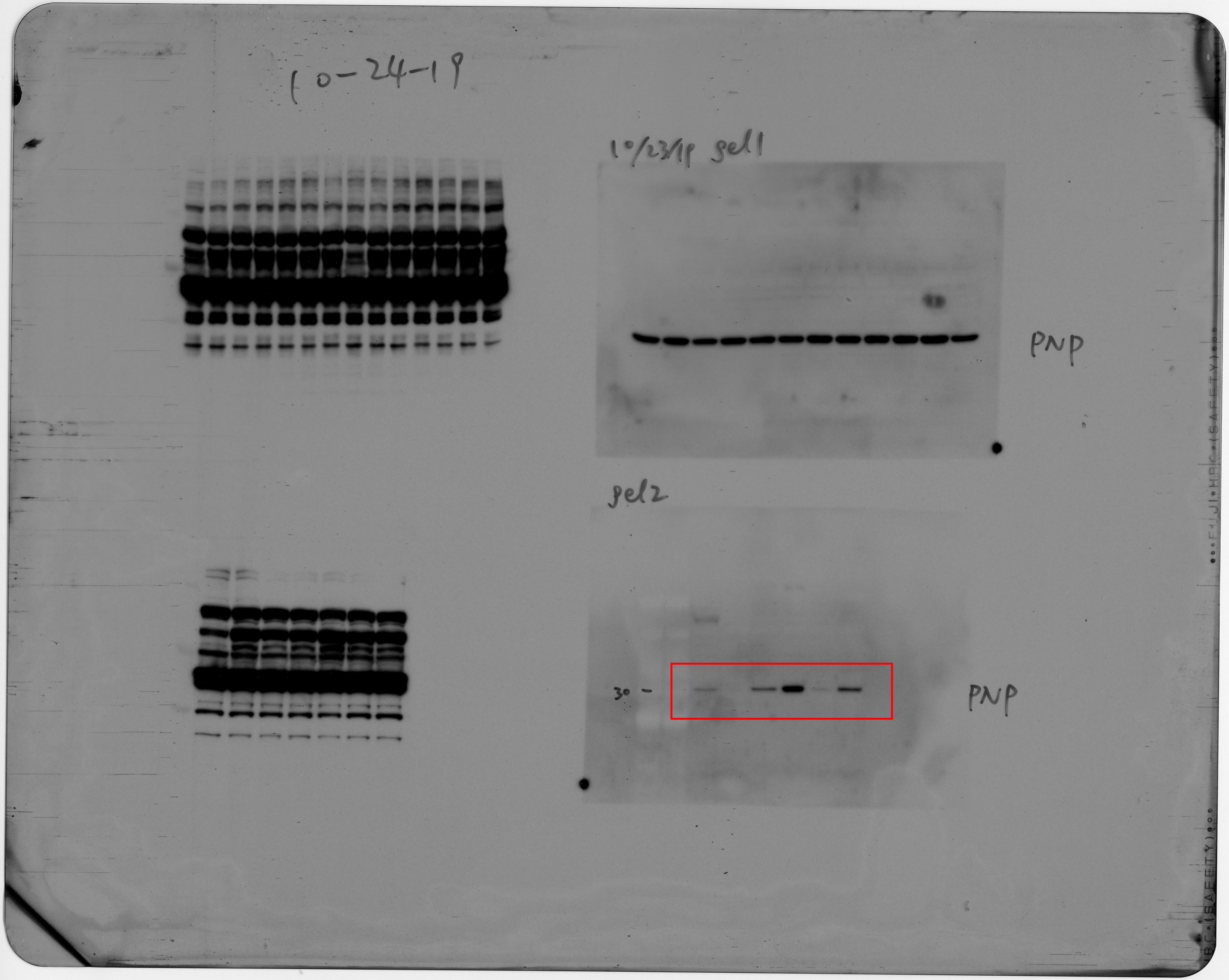
10-24-19
10/23/19 gel1
PNP
gel2
30 -
PNP

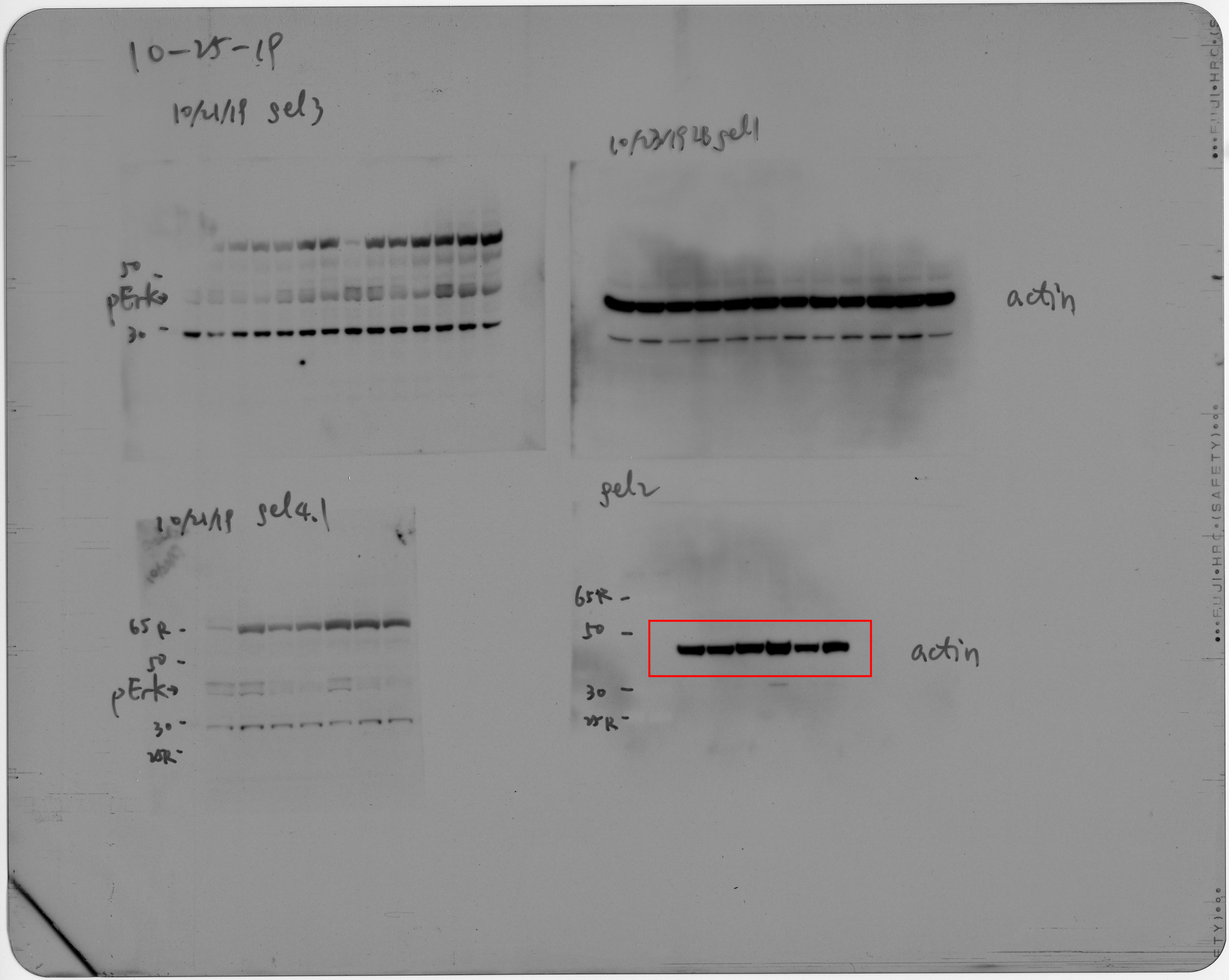

10-25-19
10/21/19 gel3
50
pErk
30
actin
gel2
10/21/19 gel4.1
65R
50
pErk
30
25R
65R
50
actin
30
25R

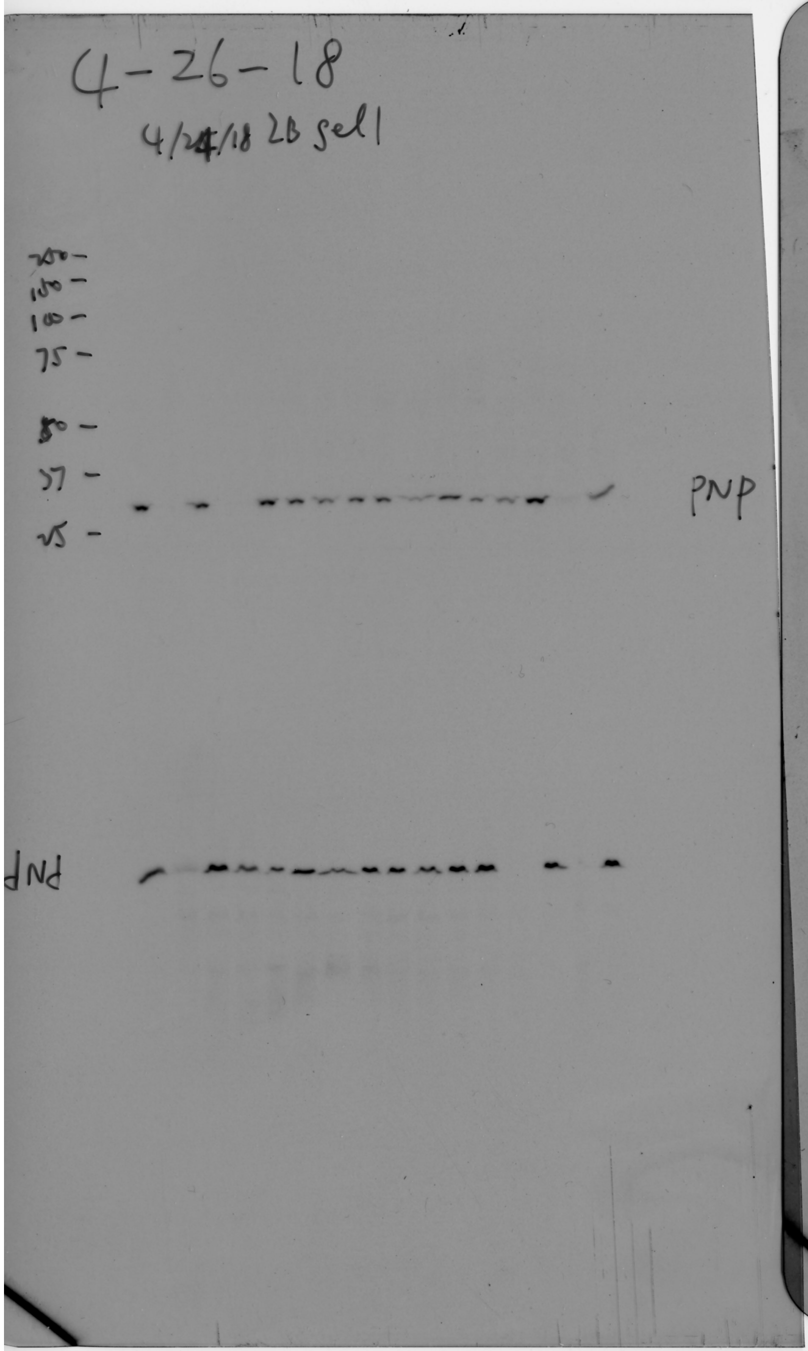

4-26-18
4/24/18 2B gel 1
250
150
100
75
50
37
25
PNP
PNP

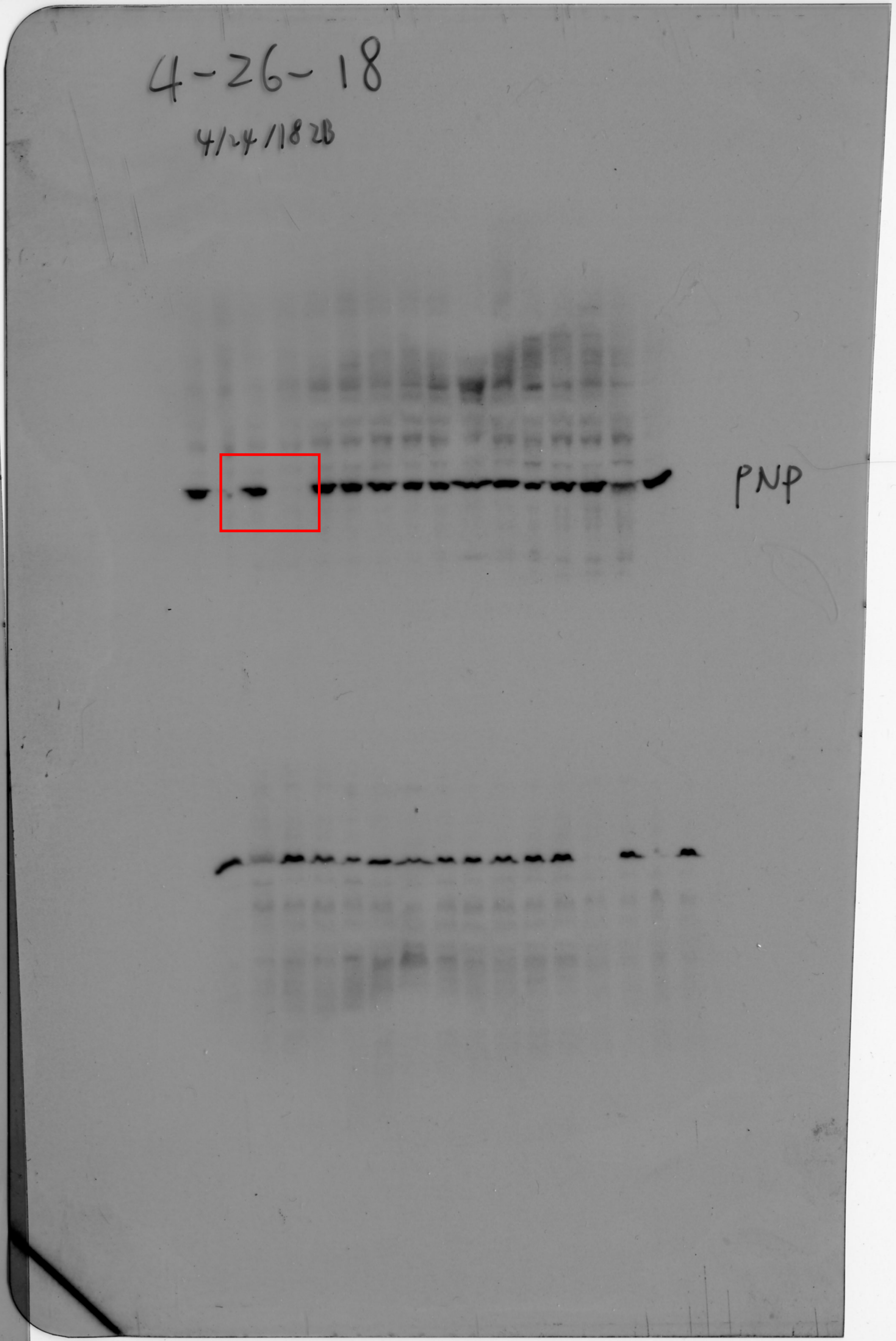

4-26-18
4/24/18 2B
PNP

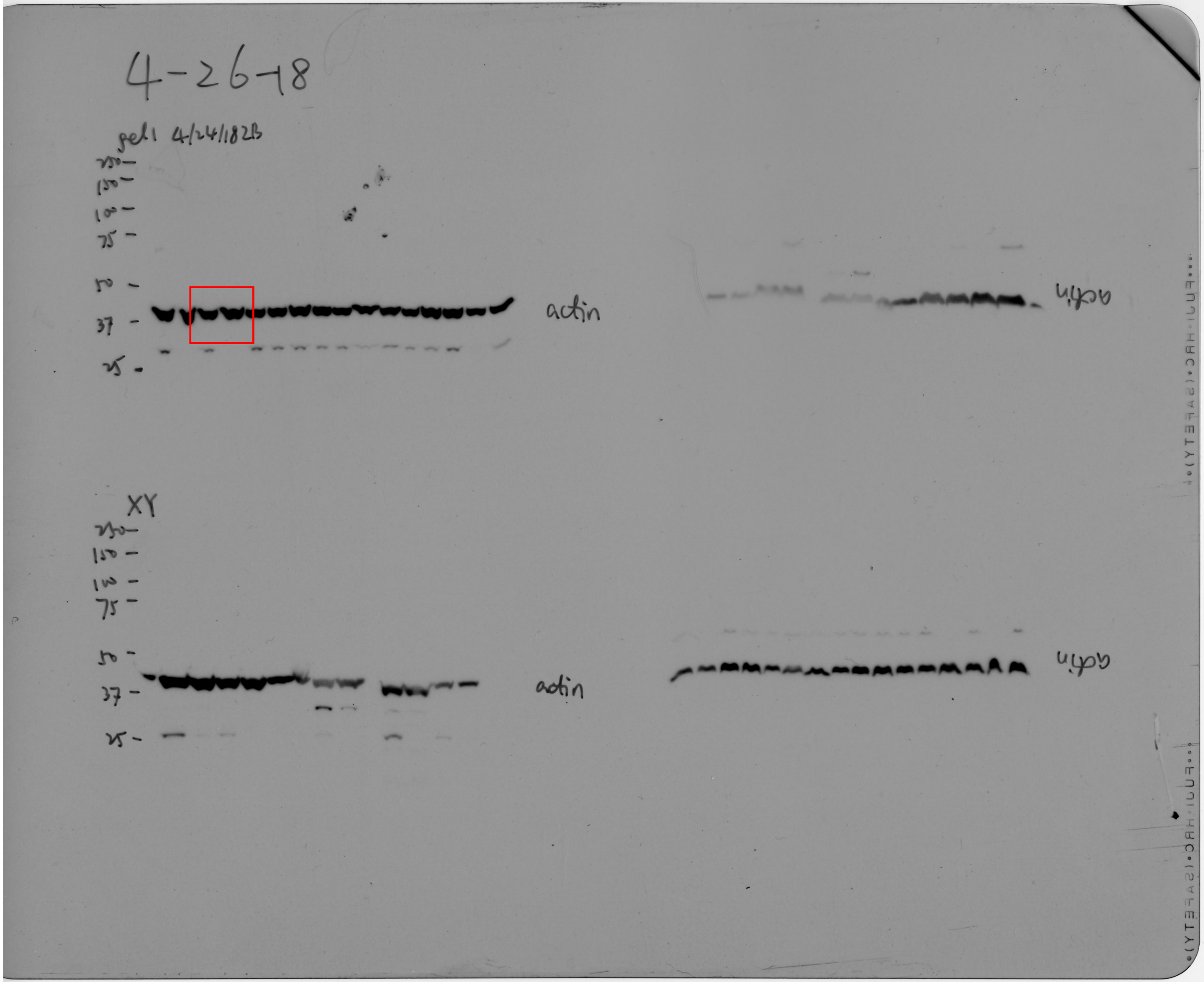
4-26-18
gel1 4/24/18 2B
250
150
100
75
50
37
25
actin
XY
250
150
100
75
50
37
25
actin
